# Hangeshashinto Induces Apoptosis and Suppresses Colorectal Cancer Growth In Vitro and In Vivo

**DOI:** 10.3390/ijms27209310

**Published:** 2026-10-20

**Authors:** Yunhai Lin, Jingya Lu, Ailing Hu, Takuji Yamaguchi, Yasushi Ikarashi, Yoshinao Harada, Kaho Ohki, Takumi Iwasawa, Hiroyuki Kobayashi

**Affiliations:** 1 Department of Personalized Kampo Medicine, Graduate School of Medicine, Juntendo University, Tokyo 113-8421, Japan; j.lu.pv@juntendo.ac.jp (J.L.); ailing@juntendo.ac.jp (A.H.); tkyamagu@juntendo.ac.jp (T.Y.); y.ikarashi.zi@juntendo.ac.jp (Y.I.); yo-harad@juntendo.ac.jp (Y.H.); koba@juntendo.ac.jp (H.K.); 2 Department of Neurosurgery, Faculty of Medicine, Juntendo University, Tokyo 113-8421, Japan; 3 Department of Nutrition Sciences, Graduate School of Health and Sports Sciences, Toyo University, Tokyo 115-8650, Japan; ohki376@toyo.jp; 4 Institution of Life Innovation Studies, Toyo University, Tokyo 115-8650, Japan; iwasawa@toyo.jp; 5 Shizuoka Medical Research Center for Disaster, Juntendo University Shizuoka Hospital, Shizuoka 410-2295, Japan

**Keywords:** hangeshashinto, colorectal cancer, apoptosis, 5-fluorouracil, xenograft model, kampo medicine

## Abstract

Hangeshashinto (HST) is a standardized seven-herb Kampo pharmaceutical used to manage gastrointestinal symptoms and chemotherapy-associated mucosal toxicity, but its direct antitumor activity remains insufficiently characterized. We examined the effects of HST in DLD-1 and SW480 colorectal cancer (CRC) cells and in non-malignant fibroblast and colonic epithelial cell models. Cell viability, colony formation, apoptosis-related endpoints, gene expression, responses to 5-fluorouracil (5-FU), and the activity of representative constituents were evaluated. Oral antitumor efficacy and tolerability were assessed in female BALB/c-nu mice bearing DLD-1 xenografts. HST preferentially reduced CRC cell viability and colony formation and increased annexin V positivity, caspase-3 activity, cleaved caspase-3 staining, and apoptotic nuclear changes. z-VAD-FMK showed a nonsignificant tendency toward partial recovery of cell viability. Reverse transcription–quantitative polymerase chain reaction findings were exploratory; statistical significance was not assessed. Co-treatment with HST (0.3 mg/mL) and 5-FU significantly reduced cell viability compared with 5-FU alone in both DLD-1 and SW480 cells; pharmacological synergy was not evaluated. Oral HST inhibited xenograft growth; tolerability assessment was limited to body weight and serum biochemistry, with reduced serum uric acid in the high-dose group after washout. These findings provide a preclinical basis for further investigation of standardized HST in CRC.

## 1. Introduction

Colorectal cancer (CRC) remains one of the leading causes of cancer-related mortality worldwide. According to the International Agency for Research on Cancer (IARC), CRC is the third most frequently diagnosed cancer and the second leading cause of cancer-related death worldwide [1,2]. Approximately 20% of patients present with distant metastasis at diagnosis, while many others are diagnosed with locally advanced disease requiring multimodal treatment [3,4].

Current treatment strategies, including surgery, systemic chemotherapy, radiotherapy for selected rectal cancers, targeted therapy, and immunotherapy, have improved patient survival. However, treatment-related adverse effects, including gastrointestinal toxicity, myelosuppression, and peripheral neuropathy, can necessitate dose reduction or treatment discontinuation [4,5,6]. In addition, the benefits of immune checkpoint inhibitors can be limited by primary or acquired resistance [7]. Therefore, there remains an unmet need for complementary therapeutic approaches that improve treatment efficacy while maintaining patients’ quality of life.

Hangeshashinto (HST; Chinese pinyin: Banxia Xiexin Tang) is a classical Kampo formulation originally described in *Shang Han Lun* and *Jin Gui Yao Lue*. Under Japan’s national health insurance system, HST is prescribed for various gastrointestinal disorders, including gastritis, stomatitis, and diarrhea. Previous clinical studies have evaluated HST for chemotherapy-induced oral mucositis and irinotecan-associated diarrhea [8,9].

Despite these clinical applications, the direct antitumor activity of HST against CRC remains insufficiently characterized, and the underlying molecular mechanisms have not been fully elucidated. Apoptotic cell death is regulated through interconnected extrinsic and intrinsic signaling pathways, including death receptor-mediated signaling and mitochondrial pathways that converge on caspase activation [10,11]. Because resistance to apoptosis represents a hallmark of cancer progression and therapeutic failure, agents capable of modulating apoptotic pathways may provide therapeutic benefit. Recent studies have highlighted the potential of natural products as complementary anticancer approaches capable of modulating multiple cell-death-related pathways [12].

Given that fluoropyrimidines remain the cornerstone of systemic treatment for CRC, it is clinically relevant to determine whether HST influences cellular responses to 5-fluorouracil (5-FU). Furthermore, because multi-component herbal formulations may preferentially affect malignant cells while minimizing toxicity to normal tissues, we evaluated the effects of HST in both CRC cells and non-malignant cell models.

In the present study, we employed complementary in vitro and in vivo approaches to investigate the antitumor activity of HST in human CRC cell lines (DLD-1 and SW480), together with its effects on normal fibroblasts and non-malignant colonic epithelial cells. We further examined apoptosis-associated cellular responses, changes in cellular responses to 5-FU, and the activity of representative HST constituents. In addition, oral antitumor efficacy and tolerability were evaluated using a nude mouse xenograft model. We hypothesized that HST would inhibit CRC growth, with apoptosis contributing to its effects.

## 2. Results

**Figure 1:**
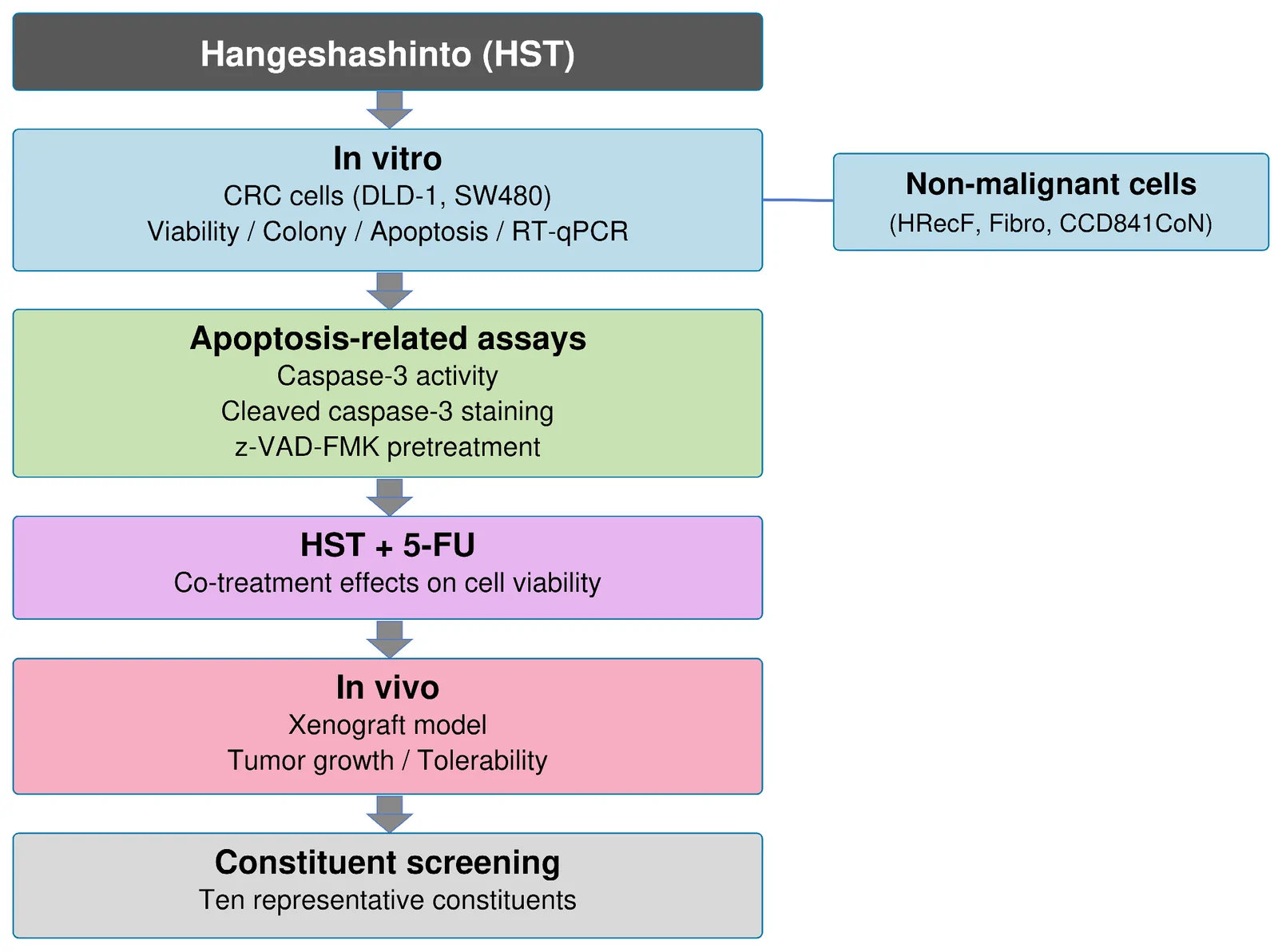
Schematic overview of the study design. The workflow begins with in vitro assays assessing cell viability, colony formation, apoptosis, and apoptosis-related gene expression, followed by analyses of apoptosis-associated responses. Combination studies with 5-fluorouracil (5-FU), in vivo xenograft experiments evaluating antitumor efficacy and tolerability, and screening of representative constituents are also included. Arrows indicate the flow of the study, and the box colors distinguish the experimental components. Abbreviations: HST, Hangeshashinto; CRC, colorectal cancer; RT-qPCR, reverse transcription–quantitative polymerase chain reaction; HRecF, human rectal fibroblasts; Fibro, adult human dermal fibroblasts; z-VAD-FMK, benzyloxycarbonyl-Val-Ala-Asp-fluoromethylketone (a pan-caspase inhibitor); 5-FU, 5-fluorouracil. DLD-1 and SW480 are human colorectal cancer cell lines.

An overview of the experimental design is presented in Figure 1.

### 2.1. Chemical Fingerprinting of HST

The three-dimensional UV–HPLC chromatogram and quantitative compositional data provided by the manufacturer (Tsumura & Co., Tokyo, Japan) are presented in Supplementary Figure S1 and Supplementary Table S1, respectively, with quantitative values summarized in the latter. From Scutellariae Radix, baicalin and wogonin were prominent, with baicalein at a trace level; from Glycyrrhizae Radix, glycyrrhizin was abundant, with liquiritin at a trace level; from Coptidis Rhizoma, berberine was detected, with palmatine at a trace level; from processed ginger, 6-gingerol was identified; and Pinelliae Tuber contributed magnoflorine at a trace level (trace < 0.3 mg/g). Quantification was performed by Tsumura & Co. using external standard calibration with JP18 reference standards, and selected minor peaks were confirmed by LC/MS, demonstrating consistency with JP18 specifications.

### 2.2. Cell Viability Assay

**Figure 2:**
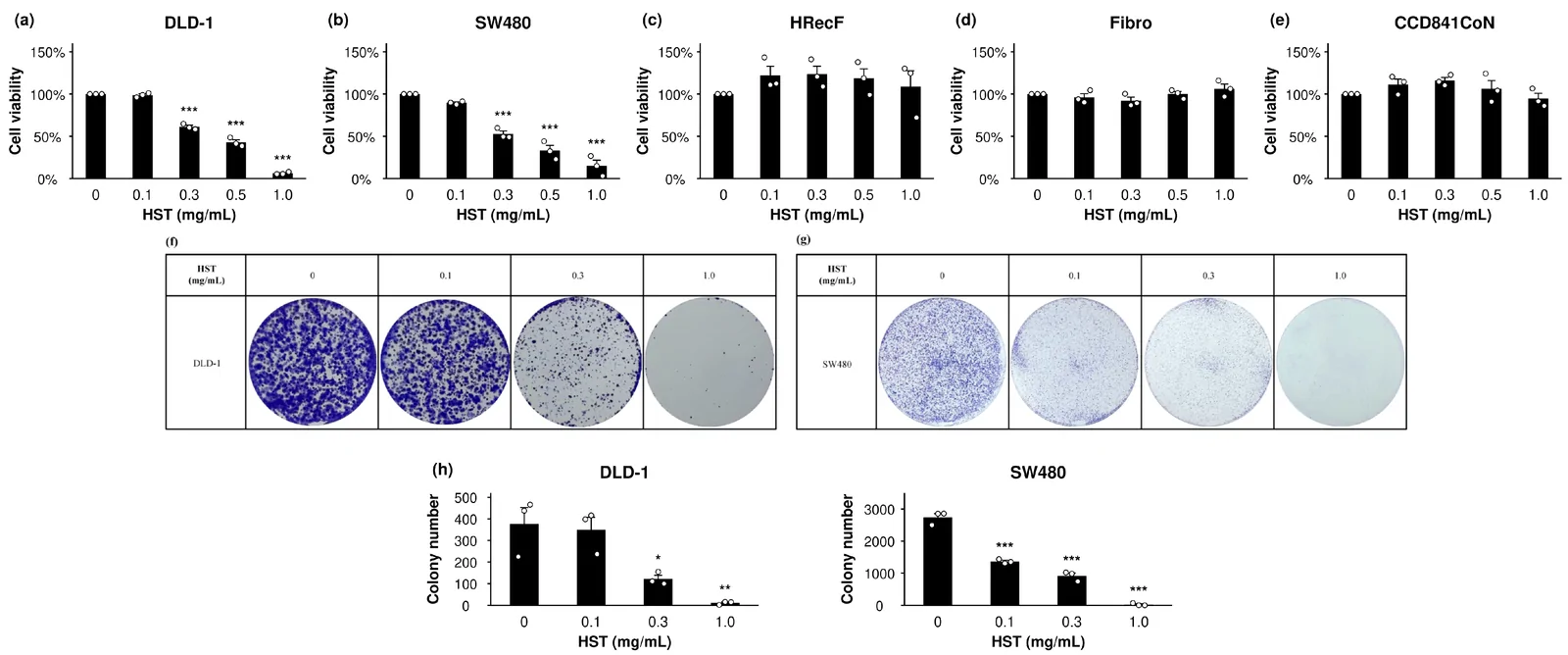
Effects of HST on cell viability and colony formation in colorectal cancer and non-malignant cells. (**a**–**e**) Cell viability of CRC cells (DLD-1 and SW480) and non-malignant cells (HRecF, Fibro, and CCD841CoN) after 48 h treatment with HST (0–1.0 mg/mL), assessed using the CCK-8 assay: (**a**) DLD-1; (**b**) SW480; (**c**) HRecF; (**d**) Fibro; (**e**) CCD841CoN. (**f**,**g**) Representative images of colony formation in DLD-1 (**f**) and SW480 (**g**) cells after 14 days of HST treatment (0–1.0 mg/mL). (**h**) Quantitative analysis of colony numbers in DLD-1 and SW480 cells using ImageJ. Data are presented as the mean ± SEM of three independent experiments (*n* = 3). Dots represent independent biological experiments. Statistical significance was determined by one-way ANOVA followed by Dunnett’s test, with the untreated control as the reference group. * *p* < 0.05, ** *p* < 0.01, *** *p* < 0.001 vs. control. Abbreviations: HST, Hangeshashinto; CRC, colorectal cancer; HRecF, human rectal fibroblasts; Fibro, adult human dermal fibroblasts; CCK-8, Cell Counting Kit-8; SEM, standard error of the mean; ANOVA, analysis of variance. DLD-1 and SW480 are human colorectal cancer cell lines; CCD841CoN is a non-malignant human colonic epithelial cell line.

We next evaluated the effects of HST on cell survival in DLD-1, SW480, HRecF, Fibro, and CCD841CoN cells (Figure 2a–e). HST caused a concentration-dependent reduction in viability in DLD-1 and SW480 cells, with the greatest reduction observed at 1.0 mg/mL. In contrast, HRecF, Fibro, and CCD841CoN cells exhibited only minimal changes in viability across the tested concentration range, with no clear evidence of cytotoxicity at the corresponding concentrations. These findings suggest that HST preferentially reduces viability in CRC cells compared with the tested non-malignant fibroblast and colonic epithelial cell models.

### 2.3. Inhibitory Effects of HST on Colony Formation

In controls, both DLD-1 (Figure 2f) and SW480 (Figure 2g) cells formed numerous large colonies, confirming robust clonogenic potential. HST treatment caused a dose-dependent reduction in colony formation, and colonies were smaller than in untreated controls. ImageJ (version 1.53) analysis showed significantly reduced colony numbers at 0.3 and 1.0 mg/mL in DLD-1 cells and at all tested concentrations (0.1, 0.3, and 1.0 mg/mL) in SW480 cells (Figure 2h). These data indicate that HST reduces the long-term proliferative capacity of CRC cells and impairs clonogenic survival in a concentration-dependent manner.

### 2.4. Apoptosis Induction (Annexin V/PI, Caspase-3 Activity, Immunofluorescence, and Nuclear Morphology)

To evaluate HST-induced apoptosis, we combined annexin V/PI flow cytometry, caspase-3 activity measurements, cleaved caspase-3 immunofluorescence, and Hoechst nuclear staining, providing complementary biochemical and morphological evidence.

#### 2.4.1. Annexin V/PI Flow Cytometric Evaluation

**Figure 3:**
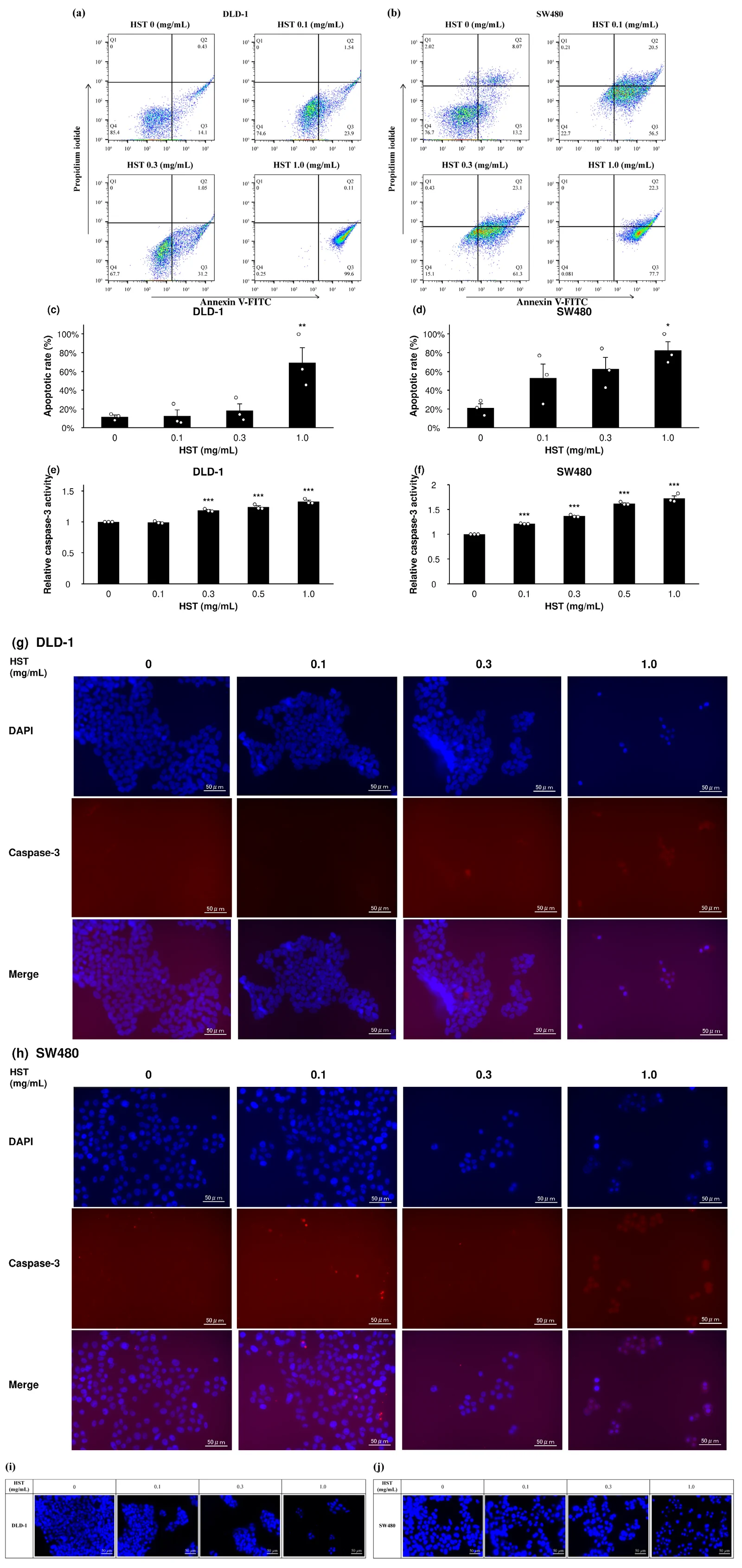
HST induces apoptosis-associated changes in colorectal cancer cells. (**a**,**b**) Representative flow cytometric dot plots of annexin V–FITC/propidium iodide staining in DLD-1 (**a**) and SW480 (**b**) human colorectal cancer cells treated with HST (0–1.0 mg/mL) for 48 h. In panels (**a**,**b**), the pseudocolor scale indicates increasing event density from blue through green and yellow to red. (**c**,**d**) Quantification of apoptotic cells (early and late apoptosis). (**e**,**f**) Caspase-3 activity measured in relative fluorescence units (RFU) after 24 h of treatment and normalized to the untreated control (=1.0). (**g**,**h**) Immunofluorescence staining of cleaved caspase-3 (red) with DAPI (blue). (**i**,**j**) Hoechst staining showing nuclear condensation and fragmentation. Data are presented as mean ± SEM from three independent biological experiments (*n* = 3). Dots in panels (**c**–**f**) represent independent biological experiments. Statistical significance was determined by one-way ANOVA followed by Dunnett’s test. * *p* < 0.05, ** *p* < 0.01, *** *p* < 0.001 vs. control. Scale bar = 50 µm (**g**–**j**). Abbreviations: HST, Hangeshashinto; FITC, fluorescein isothiocyanate; DAPI, 4′,6-diamidino-2-phenylindole; RFU, relative fluorescence units; SEM, standard error of the mean; ANOVA, analysis of variance.

DLD-1 and SW480 cells were treated with HST (0, 0.1, 0.3, 1.0 mg/mL) for 48 h and analyzed by annexin V–FITC/PI flow cytometry (Figure 3a–d). DLD-1 cells (Figure 3a,c) showed a concentration-dependent increase in the total apoptotic fraction (early and late apoptosis), with a marked elevation at the highest concentration. SW480 cells (Figure 3b,d) exhibited a similar concentration-dependent increase in apoptosis. The total apoptotic fraction was significantly increased at 1.0 mg/mL in both cell lines compared with untreated controls (Figure 3c,d), consistent with the other apoptosis-related findings.

#### 2.4.2. Caspase-3 Activity Assay

Caspase-3 activity was measured fluorometrically in DLD-1 and SW480 cells treated with HST (0–1.0 mg/mL) for 24 h (Figure 3e,f). HST increased caspase-3 activity in a concentration-dependent manner in both cell lines. Caspase-3 activity, measured in relative fluorescence units (RFU) and normalized to the untreated control (=1.0), was significantly increased at HST concentrations of ≥0.3 mg/mL in DLD-1 cells and ≥0.1 mg/mL in SW480 cells. These findings support HST-associated caspase-3 activation, consistent with the other apoptosis-related findings.

#### 2.4.3. Cleaved Caspase-3 Immunofluorescence Assay

Cleaved caspase-3 immunofluorescence was used to visualize HST-induced apoptosis in DLD-1 (Figure 3g) and SW480 (Figure 3h) cells. After 48 h of treatment with HST (0, 0.1, 0.3, 1.0 mg/mL), cells were stained with anti-cleaved caspase-3 and DAPI. DAPI labeled nuclei in blue, whereas cleaved caspase-3-positive cells appeared red. Control cells showed intact nuclei with minimal red signal, whereas HST produced a progressive, dose-dependent increase in cleaved caspase-3-positive cells. At 1.0 mg/mL, numerous cells exhibited intense red fluorescence with condensed nuclei, consistent with increased caspase-3 activation. These findings support the involvement of dose-dependent apoptosis induction, accompanied by caspase-3 activation.

#### 2.4.4. Nuclear Morphology Staining Assay

DLD-1 (Figure 3i) and SW480 (Figure 3j) cells were treated with HST (0, 0.1, 0.3, 1.0 mg/mL) for 48 h and stained with Hoechst 33258 (1 μg/mL). Both lines showed a concentration-dependent progression of nuclear changes. At 0.3 mg/mL, many cells exhibited chromatin condensation and increased fluorescence; at 1.0 mg/mL, pronounced nuclear condensation and fragmentation were evident, consistent with apoptotic morphology. These qualitative observations corroborate the annexin V/PI and cleaved caspase-3 data and further support HST-induced apoptosis.

### 2.5. Effects of z-VAD-FMK on HST-Induced Reduction in Cell Viability

**Figure 4:**
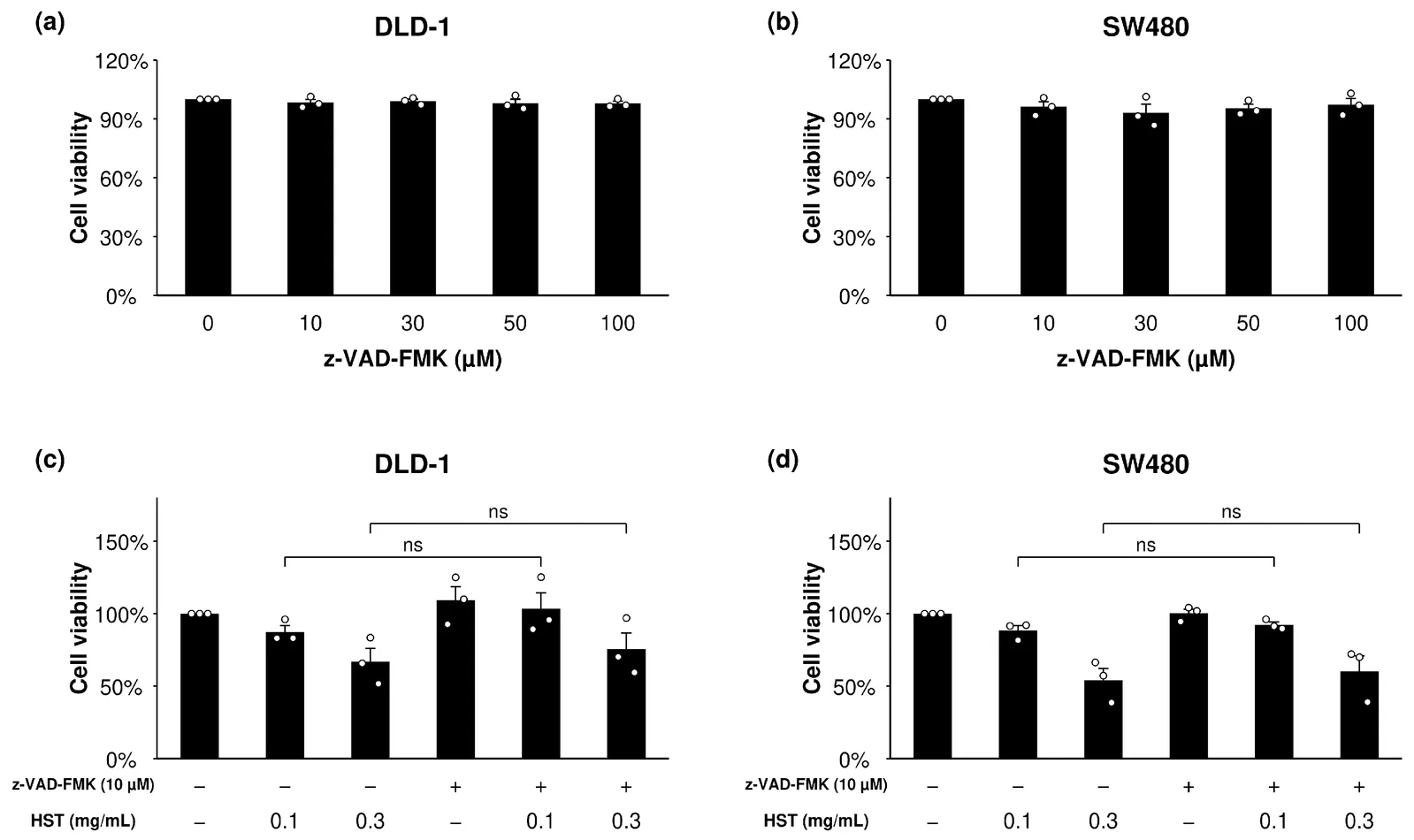
Effects of the pan-caspase inhibitor z-VAD-FMK on HST-associated reductions in cell viability in colorectal cancer cells. (**a**,**b**) Effects of z-VAD-FMK alone (0–100 μM, 48 h) on cell viability in DLD-1 (**a**) and SW480 (**b**) cells. (**c**,**d**) Effects of z-VAD-FMK pretreatment (10 μM, 30 min) on cell viability in DLD-1 (**c**) and SW480 (**d**) cells subsequently treated with HST (0.1 or 0.3 mg/mL) for 48 h. Cell viability was numerically higher following z-VAD-FMK pretreatment than with HST alone; however, none of the direct comparisons between HST alone and HST plus z-VAD-FMK at the corresponding HST concentrations reached statistical significance (all *p* > 0.05). Data are presented as mean ± SEM from three independent experiments (*n* = 3). Dots represent independent biological experiments. Brackets indicate comparisons between HST alone and HST plus z-VAD-FMK at the corresponding HST concentrations; ns, not significant. Statistical comparisons were performed using one-way ANOVA followed by Tukey’s multiple comparisons test. Abbreviations: HST, Hangeshashinto; z-VAD-FMK, benzyloxycarbonyl-Val-Ala-Asp-fluoromethylketone (a pan-caspase inhibitor); SEM, standard error of the mean; ANOVA, analysis of variance. DLD-1 and SW480 are human colorectal cancer cell lines.

The pan-caspase inhibitor z-VAD-FMK was used to assess the contribution of caspases to the HST-induced reduction in cell viability. z-VAD-FMK alone (0–100 μM, 48 h) did not significantly affect cell viability in DLD-1 or SW480 cells (Figure 4a,b). Preincubation with z-VAD-FMK (10 μM, 30 min) followed by HST (0.1 or 0.3 mg/mL, 48 h) showed a tendency toward partial recovery of cell viability in both cell lines (Figure 4c,d). However, none of the direct comparisons between HST alone and HST combined with z-VAD-FMK at the same HST concentration reached statistical significance. Thus, this experiment suggests a possible contribution of caspase-dependent processes but does not establish caspase dependence; other mechanisms may also contribute to the HST-induced reduction in cell viability.

### 2.6. RT-qPCR Analysis

To examine the transcriptional effects of HST on apoptosis-related genes, DLD-1 and SW480 cells were treated with HST (0, 0.05, 0.1, and 0.3 mg/mL) for 48 h and analyzed by RT-qPCR (Supplementary Figure S2a–f for DLD-1 and Supplementary Figure S2g–l for SW480). In both cell lines, mean *BCL2* expression was lower following HST treatment, whereas *BAX* and *TNFRSF10B* showed variable responses depending on concentration and cell line. *TNFRSF10A* showed only minor changes. Mean *BBC3* expression was higher at lower concentrations and lower at the highest concentration in SW480 cells but remained largely unchanged in DLD-1 cells. *TP53* expression showed no consistent concentration-dependent pattern across the two cell lines. Overall, the observed expression patterns are presented descriptively as exploratory findings. Statistical significance was not assessed, and these results should be interpreted cautiously given the limited number of independent experiments and inter-experimental variability; they do not establish activation of a specific apoptotic pathway.

### 2.7. Effects of Combined HST and 5-Fluorouracil Treatment on Cell Viability

**Figure 5:**
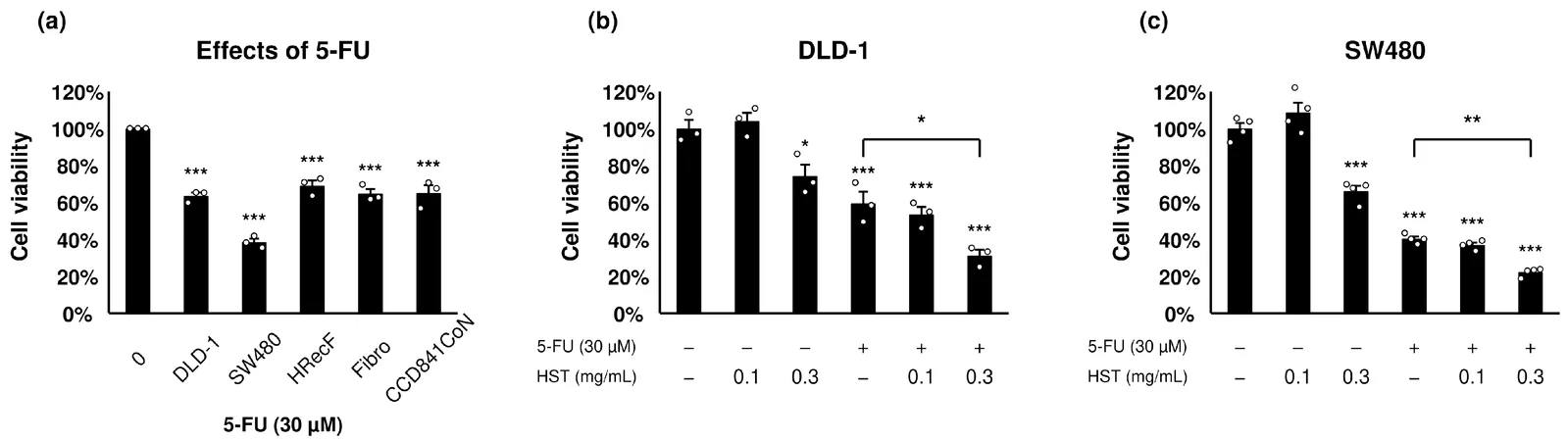
Effects of HST and 5-FU, alone and in combination, on cell viability. (**a**) Effects of 5-FU (30 μM) on cell viability in colorectal cancer cells (DLD-1 and SW480) and non-malignant cell models (HRecF, Fibro, and CCD841CoN). The bar labeled “0” represents the untreated control, normalized to 100%. (**b**,**c**) Effects of HST (0.1 or 0.3 mg/mL), 5-FU (30 μM), and their combinations on cell viability in DLD-1 (**b**) and SW480 (**c**) cells. Cell viability was assessed after 48 h using the CCK-8 assay. Data are presented as mean ± SEM (*n* = 3 for panels (**a**) and (**b**); *n* = 4 for panel (**c**)). Dots represent independent biological experiments. Statistical comparisons were performed separately for each cell model using one-way ANOVA followed by Tukey’s multiple comparisons test. For panel (**a**), the analysis included the untreated control and all tested 5-FU concentrations (10, 30, 50, and 100 μM); only the comparisons between the untreated control and 30 μM 5-FU are shown. Asterisks above individual bars indicate comparisons with the corresponding untreated control. Brackets in panels (**b**,**c**) indicate comparisons between 5-FU alone and 5-FU plus HST (0.3 mg/mL). * *p* < 0.05, ** *p* < 0.01, *** *p* < 0.001. Abbreviations: HST, Hangeshashinto; 5-FU, 5-fluorouracil; CCK-8, Cell Counting Kit-8; SEM, standard error of the mean; ANOVA, analysis of variance; HRecF, human rectal fibroblasts; Fibro, adult human dermal fibroblasts. DLD-1 and SW480 are human colorectal cancer cell lines; CCD841CoN is a non-malignant human colonic epithelial cell line.

The effect of 5-FU monotherapy was first assessed in DLD-1, SW480, HRecF, Fibro, and CCD841CoN cells (Figure 5a). To examine the effects of co-treatment with HST and 5-FU on cell viability in CRC cells, combination assays were subsequently performed in DLD-1 and SW480 cells (Figure 5b,c). A 48 h exposure to 30 μM 5-FU reduced cell viability in both DLD-1 and SW480 cells and served as the baseline for comparison. Co-treatment with HST at 0.3 mg/mL significantly reduced cell viability compared with 5-FU alone in both DLD-1 and SW480 cells (Figure 5b,c; Tukey-adjusted *p* = 0.0144 and 0.0034, respectively).

### 2.8. In Vivo Antitumor Efficacy

**Figure 6:**
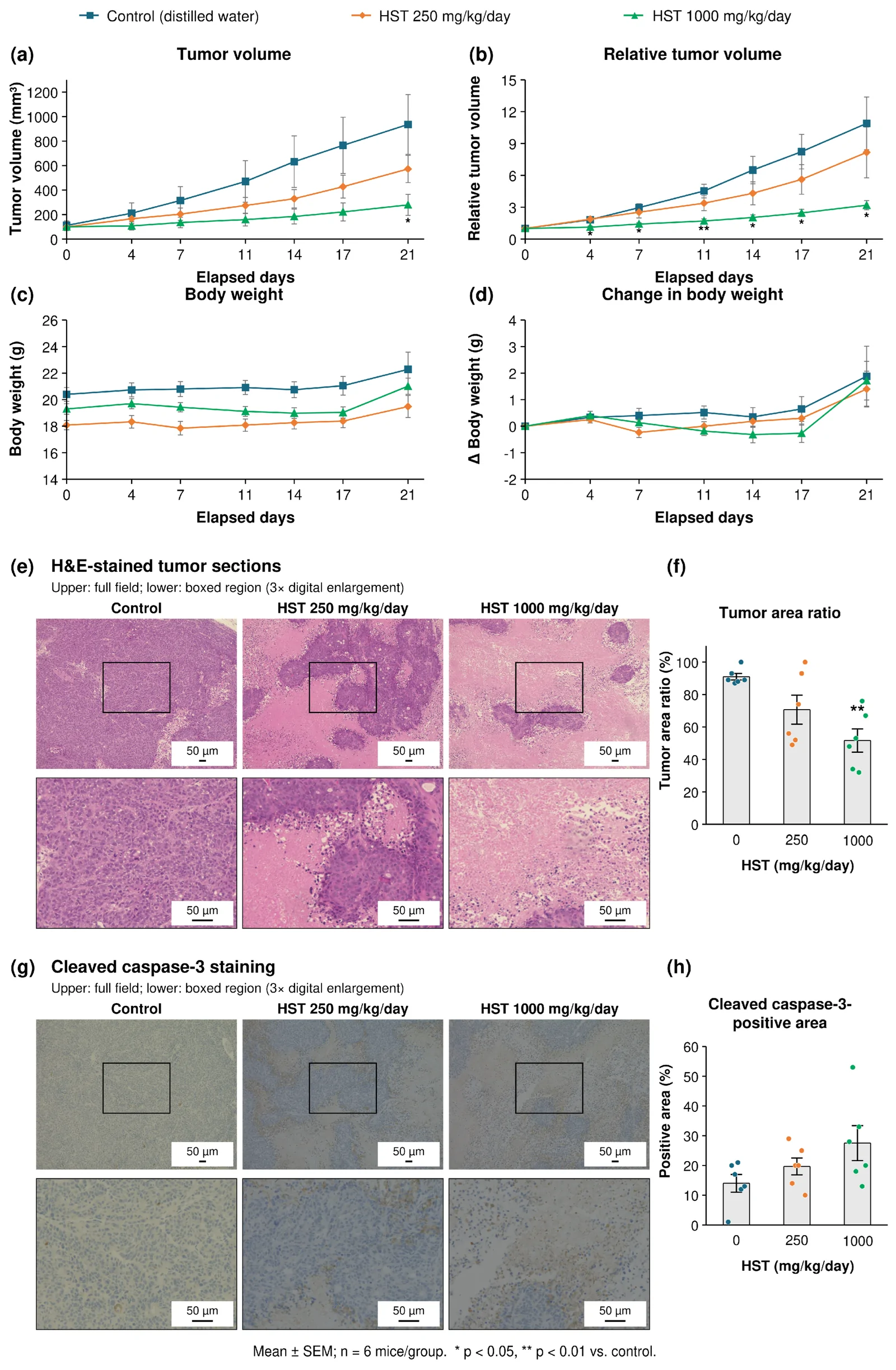
In vivo antitumor efficacy and tolerability of Hangeshashinto (HST) in a DLD-1 xenograft mouse model. (**a**) Tumor volume; (**b**) relative tumor volume normalized to each mouse’s baseline value; (**c**) body weight; (**d**) change in body weight from baseline; (**e**) representative hematoxylin and eosin (H&E)-stained tumor sections; (**f**) tumor area ratio; (**g**) representative cleaved caspase-3 immunohistochemical staining; (**h**) cleaved caspase-3-positive area. HST was administered orally at 250 or 1000 mg/kg/day for 14 days, followed by a 7-day washout period; controls received distilled water. Data are presented as mean ± SEM (*n* = 6 mice/group); dots in (**f**,**h**) represent individual mice. Longitudinal data (**a**–**d**) were analyzed using two-way repeated-measures ANOVA with Greenhouse–Geisser correction for time and group-by-time effects. Baseline values were excluded from analyses of relative tumor volume and changes in body weight. Tumor volume and relative tumor volume were additionally compared among the three groups at each post-baseline time point using one-way ANOVA followed by Tukey’s test. Multiplicity adjustment covered the three pairwise comparisons within each time point, without adjustment across time points. Endpoint data (**f**,**h**) were analyzed using one-way ANOVA followed by Dunnett’s test. * *p* < 0.05, ** *p* < 0.01 vs. control; annotations in (**a**,**b**) indicate high-dose HST versus control. In (**e**,**g**), the lower images show threefold digital enlargements of the boxed regions in the upper images. All scale bars represent 50 µm. Abbreviations: HST, Hangeshashinto; H&E, hematoxylin and eosin; SEM, standard error of the mean; ANOVA, analysis of variance.

HST was administered orally at 250 or 1000 mg/kg/day for 14 days, followed by a 7-day washout period, while control mice received distilled water. The high-dose group showed a slower increase in tumor volume than controls. Significant group-by-time interactions were observed for tumor volume and relative tumor volume (Figure 6a,b; Greenhouse–Geisser-corrected *p* = 0.0154 and 0.0490, respectively). In exploratory time-point analyses using one-way ANOVA followed by Tukey’s test, the high-dose group showed significantly lower tumor volume than controls on day 21 (Figure 6a). Relative tumor volume in the high-dose group was significantly lower than in controls on days 4, 7, 11, 14, 17, and 21 (Figure 6b). These comparisons were adjusted within each time point, without adjustment across time points. Body weight and changes in body weight are shown in Figure 6c,d.

Histopathological analysis of tumor tissues collected after the 7-day washout period (Figure 6e) revealed dense tumor cell proliferation with nuclear atypia in the control group, whereas HST-treated tumors showed reduced cellular density and areas suggestive of necrosis. Quantitative analysis demonstrated reduced tumor area ratios in the HST-treated groups (Figure 6f), with a significant reduction in the high-dose group compared with controls (Dunnett-adjusted *p* = 0.0017). Cleaved caspase-3 immunostaining (Figure 6g,h) showed a tendency toward an increased positive area, but the differences did not reach statistical significance.

### 2.9. In Vivo Tolerability Evaluation

To evaluate tolerability, body weight was monitored during the 14-day oral HST treatment and subsequent 7-day washout period, and serum biochemical parameters were assessed following the washout period. Changes in body weight from baseline did not differ significantly among groups (Figure 6d), although baseline body weight differed among groups (Figure 6c). Serum aspartate aminotransferase (AST), alanine aminotransferase (ALT), blood urea nitrogen (BUN), and creatinine (CRE) levels showed no significant differences between control and HST-treated groups (Table 1). Uric acid (UA) levels were significantly lower in the 1000 mg/kg/day group than in controls (Dunnett-adjusted *p* = 0.0274). These findings describe tolerability within the scope of body weight monitoring and the measured serum biochemical parameters.

### 2.10. Effects of Representative HST Constituents on Cell Viability

**Figure 7:**
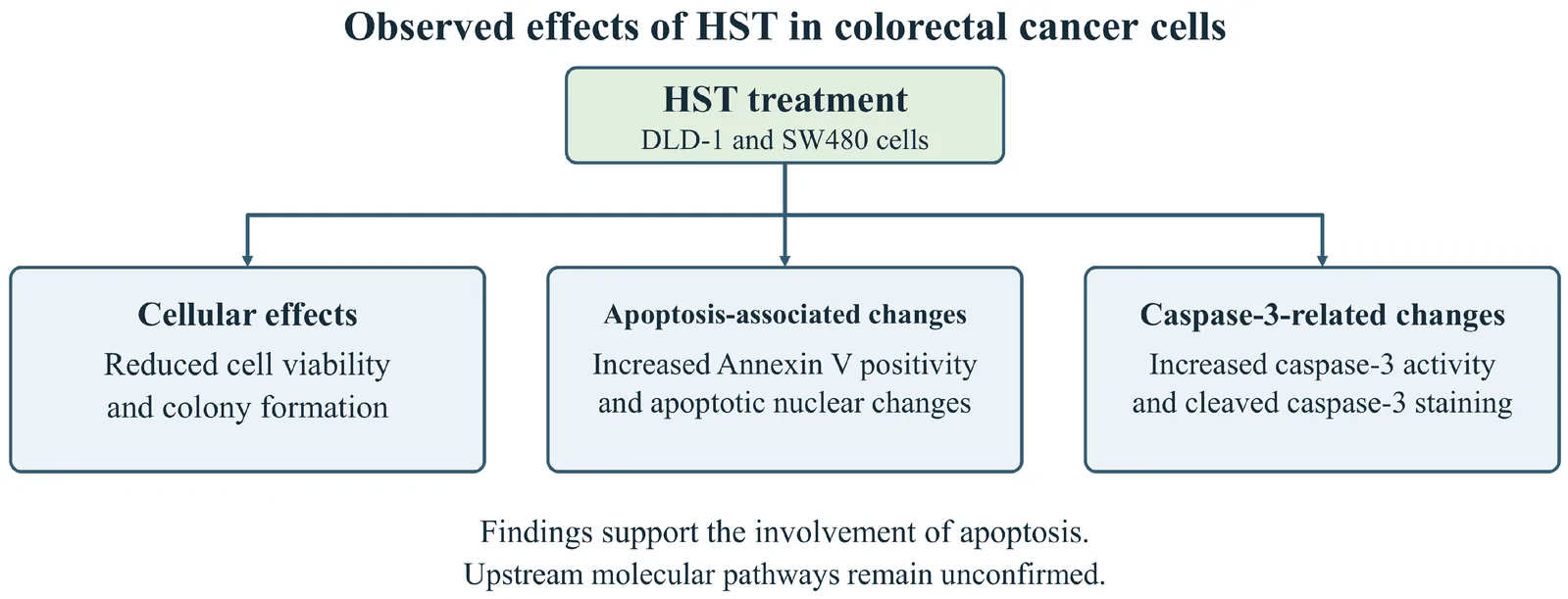
Summary of the observed effects of HST in colorectal cancer cells. HST treatment reduced cell viability and colony formation and increased annexin V positivity, apoptotic nuclear changes, caspase-3 activity, and cleaved caspase-3 staining in DLD-1 and SW480 cells. These findings support the involvement of apoptosis but do not establish the upstream molecular pathways. Arrows indicate effects observed following HST treatment rather than confirmed molecular interactions. Abbreviation: HST, Hangeshashinto.

Ten representative constituents—baicalein, wogonin, baicalin, berberine, 6-gingerol, ginsenoside Rg1, ginsenoside Rb1, isoliquiritigenin, liquiritin, and glycyrrhizic acid—were evaluated for their effects on cell viability after 48 h (Supplementary Figure S3). The estimated IC_50_ values and growth inhibition profiles are summarized in Supplementary Table S2. Baicalein and wogonin significantly reduced cell viability in both DLD-1 and SW480 cells at all tested concentrations (12.5–100 μM) compared with vehicle controls (Supplementary Figure S3). Baicalin significantly reduced viability at ≥25 μM in DLD-1 and ≥50 μM in SW480 cells. Berberine significantly reduced cell viability in SW480 cells at 12.5–100 μM, whereas no significant reduction was observed in DLD-1 cells. A clear decrease in DLD-1 cell viability was observed only at 100 μM isoliquiritigenin, but not in SW480. In contrast, 6-gingerol, ginsenoside Rg1/Rb1, liquiritin, and glycyrrhizic acid had no significant effects in either cancer cell line (*p* ≥ 0.05). In Fibro cells, wogonin significantly reduced cell viability, whereas isoliquiritigenin showed a numerical reduction without statistical significance; the other tested compounds showed little or no inhibitory effect. A schematic summary of the observed effects of HST in colorectal cancer cells is shown in Figure 7.

## 3. Discussion

This study showed that HST exerted dose-dependent growth inhibition in DLD-1 and SW480 cells while exerting smaller effects on HRecF, Fibro, and CCD841CoN cells. These findings indicate preferential effects on CRC cells under the experimental conditions tested, but do not establish tumor-selective cytotoxicity, because two of the non-malignant models were fibroblasts and only one was a non-malignant colonic epithelial cell line. Although relatively high concentrations of HST (up to 1.0 mg/mL) were used in vitro, these exploratory exposures were not intended to approximate clinically achievable plasma concentrations. Therefore, further pharmacokinetic/pharmacodynamic (PK/PD) and dose-translation studies are required to determine clinically relevant exposure levels. The present study combines complementary apoptosis-related assays, exploratory HST/5-FU co-treatment experiments, constituent screening, and oral xenograft testing, extending the evaluation of HST beyond its established supportive-care applications.

Multiple apoptosis-related endpoints—including annexin V positivity, caspase-3 activation, cleaved caspase-3 immunofluorescence, and nuclear condensation—suggest that apoptosis contributes to the effects of HST. However, z-VAD-FMK did not significantly restore cell viability compared with HST alone, so caspase dependence remains unconfirmed. Exploratory RT-qPCR showed lower mean *BCL2* expression and variable *BAX* and *TNFRSF10B* responses, without inferential statistical testing. These mRNA findings do not establish protein changes or pathway activation, including DR5 signaling [13]. DLD-1 and SW480 harbor reported *TP53* mutations [14]; p53-independent PUMA regulation involving Sp1/p73 [15] and the context-dependent roles of p73 [16] provide background but were not investigated here. Upstream apoptotic pathways and AIF-associated caspase-independent cell death [17] were not examined. Figure 7 therefore summarizes observed effects rather than confirmed molecular mechanisms. Reduced proliferation may contribute to the decrease in cell viability [18], while other stress or death responses remain possible [10]; future studies measuring DNA synthesis using 5-ethynyl-2′-deoxyuridine (EdU) [19] could clarify antiproliferative effects.

Previous studies provide relevant context for the constituent screening results. Dou et al. reported that baicalein and baicalin inhibited colorectal cancer cell growth through apoptosis and senescence, respectively, indicating that related flavonoids can produce distinct cellular responses [20]. Kim et al. observed apoptosis-associated changes and reduced viability following baicalein or wogonin treatment in HT-29 cells [21]. These findings are broadly consistent with the inhibitory activities of these compounds in the present screening, but do not demonstrate that the whole HST preparation acts through the same mechanisms. Previous work also showed that 6-gingerol induced growth arrest and apoptosis in colorectal cancer cells, including experiments using 200 μM for 72 h [22]. In contrast, 6-gingerol did not significantly reduce viability in our DLD-1 and SW480 assays at concentrations up to 100 μM after 48 h. Differences in concentration, exposure duration, and cell model may contribute to these differing observations. Berberine reduced cell viability in SW480 cells but showed no significant reduction in DLD-1 cells, suggesting cell-line-dependent responses under the tested conditions.

The relationship between the isolated-compound concentrations and their abundance in HST is important when interpreting these findings. Based on the quantitative composition in Supplementary Table S1, HST at 1.0 mg/mL would provide approximately 37.6 μM baicalin, 6.7 μM wogonin, 2.7 μM berberine, 1.7 μM 6-gingerol, and less than 1.1 μM baicalein. The isolated compounds were tested at 12.5–100 μM; thus, particularly for baicalein and wogonin, the screening concentrations exceeded those supplied by the extract. Activity at these higher concentrations does not establish a substantial contribution to the effects of the whole preparation. Accordingly, baicalein, baicalin, and wogonin should be regarded as compounds showing activity in the screening rather than established principal bioactive constituents of HST. Interactions among constituents and their exposure following oral administration were not assessed and require further investigation.

Previous studies have also investigated *Scutellaria baicalensis* and baicalin in combination with 5-FU, reporting enhanced antitumor effects associated with inhibition of proliferative signaling, including the cyclin-dependent kinase–retinoblastoma protein (CDK–RB) pathway [23]. These findings provide a rationale for studying herbal preparations alongside 5-FU, but cannot be directly extrapolated to HST because the preparations, constituent exposures, and experimental models differ. In the present study, co-treatment with HST at 0.3 mg/mL significantly reduced cell viability compared with 5-FU alone in both DLD-1 and SW480 cells. Only one 5-FU concentration and two HST concentrations were evaluated in the combination experiments, and formal synergy modeling was not performed. The results therefore describe co-treatment effects under the tested conditions and do not establish synergy or chemosensitization.

Oral HST suppressed DLD-1 xenograft growth, and tumor suppression remained detectable after the 7-day washout period. This observation does not establish a durable antitumor response after treatment cessation. Tumor tissues also showed a tendency toward increased cleaved caspase-3 staining, but these findings do not establish the mechanism responsible for tumor suppression. Tolerability assessment was limited to body weight monitoring and serum biochemical measurements; major-organ histopathology was not sufficiently evaluated. Serum measurements were obtained after the 7-day washout period and may therefore have missed transient abnormalities during treatment. These assessments do not establish general safety. Other limitations include the use of a single xenograft model and six mice per group without an a priori statistical sample size calculation. Although allocation used block-based random assignment and tumor measurements were performed blinded to treatment allocation, the final grouping was selected after repeated random allocations based on investigator judgment of baseline balance, and histological assessments were not blinded. These limitations leave potential selection and observer bias and warrant cautious interpretation of the in vivo findings. Future studies should prioritize exposure-relevant testing, validation in organoid or patient-derived models, broader evaluation of apoptosis-related and non-apoptotic responses, and systematic analysis of HST/5-FU interactions. Collectively, the present findings support further preclinical evaluation of HST as a complementary therapeutic candidate in colorectal cancer.

## 4. Materials and Methods

### 4.1. Experimental Design

An overview of the experimental workflow is presented in Figure 1. The study employed integrated in vitro and in vivo approaches, including evaluation of the HST extract, in vitro assays, mechanistic analyses, in vivo validation, and constituent screening. The quantitative component profile of HST is provided in Supplementary Table S1. Detailed information on reagents is provided in Supplementary Table S3. For in vitro assays, HST was dissolved directly in medium; for in vivo studies, it was suspended in distilled water. Therefore, DMSO was not used in HST preparation. DMSO (≤0.1%) served solely as the vehicle for isolated constituents and z-VAD-FMK.

### 4.2. Test Substance (HST)

A spray-dried extract of HST (Lot 2210014010; Tsumura & Co., Tokyo, Japan), compliant with *The Japanese Pharmacopoeia* 18th edition (JP18), was used. As this study used HST as a commercially available Kampo formulation (Tsumura & Co., Tokyo, Japan), no specific governmental permission for access to plant materials was required under the framework of the Convention on Biological Diversity. The seven-herb composition, including botanical identities and ratios, followed JP18 (Table 2). Authenticity and lot characteristics were documented using a manufacturer-provided three-dimensional HPLC (UV) chromatogram (Supplementary Figure S1). Quantitative markers are listed in Supplementary Table S1. Detection wavelengths and calibration information are provided in the notes to Supplementary Table S1, and quantitative results are presented in Section 2.1.

### 4.3. Cell Lines and Culture Settings

DLD-1 human colorectal cancer cells (JCRB9094; Cellosaurus: CVCL_0248; RRID:CVCL_0248) and the SW480-derived luciferase-expressing colorectal cancer cell line SW-480/CMV-Luc (NIHS0738; Cellosaurus: CVCL_4Y13; RRID:CVCL_4Y13) were obtained directly from the JCRB Cell Bank (Osaka, Japan). SW-480/CMV-Luc is referred to as “SW480” throughout this manuscript. Primary human rectal fibroblasts (HRecF; Cat. No. 2960) were obtained from ScienCell Research Laboratories (Carlsbad, CA, USA). Primary adult human dermal fibroblasts (designated “Fibro” in this study; Cat. No. CA106K05a) were manufactured by Cell Applications, Inc. (San Diego, CA, USA) and supplied through Toyobo Co., Ltd. (Osaka, Japan). CCD 841 CoN (hereafter referred to as CCD841CoN) cells (ATCC CRL-1790; Cellosaurus: CVCL_2871; RRID:CVCL_2871) were purchased directly from the American Type Culture Collection (ATCC; Manassas, VA, USA). Cell culture conditions for each assay are described in the corresponding Supplementary Methods sections.

### 4.4. Reagents, Antibodies, and Chemicals

A consolidated list of all reagents, media, antibodies, and chemicals with suppliers and catalog numbers is provided in Supplementary Table S3. The principal assay kits and staining reagents included Cell Counting Kit-8 (CCK-8; Dojindo Laboratories, Kumamoto, Japan; CK04-11), Hoechst 33258 staining solution (Dojindo Laboratories; H341), the Dead Cell Apoptosis Kit with Annexin V FITC & Propidium Iodide (Thermo Fisher Scientific, Waltham, MA, USA; V13242), and the Caspase-3 Activity Assay Kit (Cell Signaling Technology, Danvers, MA, USA; #5723). Antibodies included Cleaved Caspase-3 (Asp175) (5A1E) Rabbit Monoclonal Antibody (#9664S) and Anti-rabbit IgG (H + L), F(ab′)_2_ Fragment (Alexa Fluor 594 Conjugate; #8889), both from Cell Signaling Technology. Molecular assay reagents included the RNeasy Plus Mini Kit (QIAGEN, Hilden, Germany; 74134), ReverTra Ace qPCR RT Master Mix with gDNA Remover (FSQ-301), and KOD SYBR qPCR Mix (QKD-201), with the latter two supplied by Toyobo Co., Ltd. (Osaka, Japan). The handling of DMSO and preparation of the HST extract are detailed in Section 4.1.

### 4.5. Manufacturer-Provided HPLC Profile and Quantitative Marker Data for HST

In this study, a manufacturer-provided quantitative chromatographic dataset of HST based on three-dimensional high-performance liquid chromatography (3D-HPLC) (Tsumura & Co., Tokyo, Japan) was used. A representative chromatogram is shown in Supplementary Figure S1, and quantitative marker compound data are summarized in Supplementary Table S1.

### 4.6. Cell Viability Assay (CCK-8)

DLD-1, SW480, and CCD841CoN cells (5 × 10^3^/well) and HRecF and Fibro cells (3 × 10^3^/well) were seeded in 96-well plates and preincubated for 24 h. Cells were then exposed to HST (0–1.0 mg/mL) for 48 h. Cell viability was assessed using Cell Counting Kit-8 (CCK-8; Dojindo Laboratories, Kumamoto, Japan; CK04-11). Detailed procedures are described in the Supplementary Methods (Section S4.6).

### 4.7. Colony Formation Assay

DLD-1 and SW480 cells (600 cells/well) were seeded in six-well plates. After 24 h, the cells were treated with HST (0–1.0 mg/mL) for 14 days, with medium replaced every 2–3 days. Colony formation was evaluated as described in the Supplementary Methods (Section S4.7), following established protocols [24,25].

### 4.8. Apoptosis Assays

Apoptosis was evaluated in DLD-1 and SW480 cells using annexin V/PI flow cytometry [26], caspase-3 activity measurement [27], cleaved caspase-3 immunofluorescence, and nuclear morphology analysis, as detailed in the Supplementary Methods (Sections S4.8.1–S4.8.4). The assays used the Dead Cell Apoptosis Kit with Annexin V FITC and Propidium Iodide (Thermo Fisher Scientific, Waltham, MA, USA; V13242), Caspase-3 Activity Assay Kit (Cell Signaling Technology, Danvers, MA, USA; #5723), cleaved caspase-3 antibody (#9664S) and Alexa Fluor 594-conjugated anti-rabbit secondary antibody (#8889; both from Cell Signaling Technology), Hoechst 33258 (Dojindo Laboratories, Kumamoto, Japan; H341), and DAPI-containing mounting medium (Vector Laboratories, Newark, CA, USA; H-1200).

### 4.9. Pharmacological Test for Caspase Dependence

The involvement of caspases in the HST-induced reduction in cell viability was assessed using z-VAD-FMK (Promega, Madison, WI, USA; G7231), as described in the Supplementary Methods (Sections S4.9.1–S4.9.2).

### 4.10. Reverse Transcription–Quantitative Polymerase Chain Reaction (RT-qPCR) Analysis

DLD-1 and SW480 cells were treated with HST (0–0.3 mg/mL, 48 h). Expression of *BCL2* (BCL-2), *BAX*, *TNFRSF10A* (DR4), *TNFRSF10B* (DR5), *TP53* (p53), and *BBC3* (PUMA) was analyzed by RT-qPCR, with *GAPDH* as the internal control. Primer sequences are listed in Table 3. RNA extraction, reverse transcription, and qPCR were performed using the RNeasy Plus Mini Kit (QIAGEN, Hilden, Germany; 74134), ReverTra Ace qPCR RT Master Mix with gDNA Remover (FSQ-301), and KOD SYBR qPCR Mix (QKD-201; both from Toyobo Co., Ltd., Osaka, Japan), respectively. Full procedures are reported in Supplementary Methods (Section S4.10).

### 4.11. Combination Treatment with 5-Fluorouracil (5-FU)

DLD-1 and SW480 cells were treated with 5-FU (30 μM), HST (0.1–0.3 mg/mL), or both for 48 h. Cell viability was assessed using the CCK-8 assay. Details are provided in the Supplementary Methods (Section S4.11).

### 4.12. In Vivo Subcutaneous Xenograft Tumor Model

A DLD-1 xenograft model in female BALB/c-nu mice was used to assess the in vivo antitumor efficacy and tolerability of HST. Experimental procedures are described in Supplementary Methods (Sections S4.12.1–S4.12.3). All animal procedures were approved by the Institutional Animal Care and Use Committee of Toyo University (Approval No. 2024-14).

#### 4.12.1. Animals, Grouping, and Oral Administration

A total of 34 female BALB/c-nu mice were obtained from The Jackson Laboratory Japan, Inc. (Yokohama, Japan). After at least one week of acclimatization, the mice were inoculated subcutaneously with 5 × 10^6^ DLD-1 cells in Matrigel/HBSS. Fourteen days after inoculation, 16 mice with tumor volumes below 10 mm^3^ were excluded before treatment. The remaining 18 mice were allocated to three groups (*n* = 6 mice per group): vehicle control, HST 250 mg/kg/day, and HST 1000 mg/kg/day. Animals were ranked according to baseline body weight and tumor volume and arranged into six blocks of three mice with similar measurements. Within each block, random numbers generated using the RAND function in Microsoft Excel were used to assign one mouse to each group. Mean body weight and tumor volume were then checked across groups. The entire random allocation procedure was repeated several times, and a grouping judged to provide satisfactory balance in these baseline variables was selected. After treatment initiation, there were no deaths, exclusions, or missing observations, and no animals or data points were excluded from the analyses. The group size was determined with reference to a previous DLD-1 xenograft study that used six mice per group [28]; no a priori statistical sample size calculation was performed. HST was administered orally twice daily for 14 days at total daily doses of 250 or 1000 mg/kg; control mice received distilled water. The HST doses were selected with reference to a previous animal study using oral HST at 250–1000 mg/kg [29]. The 14-day treatment period was informed by a previous study using approximately two weeks of oral hochuekkito administration in tumor-bearing mice [30]. Twice-daily administration was chosen with reference to divided dosing in clinical Kampo use. Following the 14-day treatment period, mice underwent a 7-day washout period before euthanasia and necropsy. This washout period was included to monitor tumor growth during the short interval following treatment cessation.

#### 4.12.2. Tumor Volume and Body Weight Measurements

Tumor size and body weight were recorded every 3–4 days. Tumor volume was calculated as (length × width^2^)/2. Tumor dimensions were measured by an investigator blinded to treatment allocation, with animals identified by individual identification numbers. Treatment administration and tumor measurements were performed by different investigators. Tumors were collected after the 7-day washout period for histopathological examination and cleaved caspase-3 immunohistochemistry.

#### 4.12.3. Blood Collection, Euthanasia, Serum Biochemistry, and Tumor Histology

After the 7-day washout period, mice were anesthetized with 2% inhaled isoflurane. After confirming the absence of a response to a pinch stimulus, terminal blood collection (approximately 1 mL per mouse) was performed from the caudal vena cava following laparotomy. Isoflurane anesthesia was maintained throughout terminal blood collection and subsequent cervical dislocation, without recovery from anesthesia. Death was confirmed by the absence of respiration, heartbeat, and a response to a pinch stimulus. Blood samples were used for serum biochemical analysis (AST, ALT, BUN, creatinine, and uric acid [UA]). Tumors were fixed in 10% neutral-buffered formalin, paraffin-embedded, sectioned, and stained with hematoxylin and eosin (H&E). Cleaved caspase-3 staining in tumors was assessed by immunohistochemistry (Cell Signaling Technology, #9664S; 1:200). Histopathological evaluation of H&E-stained sections and quantification of cleaved caspase-3 staining were performed with knowledge of the treatment assignments and were not blinded. Serum biochemical parameters were evaluated based on intergroup comparisons rather than predefined reference ranges.

### 4.13. Screening of Isolated Constituents

Ten representative constituents of HST were dissolved in DMSO to prepare stock solutions and diluted to final concentrations of 12.5, 25, 50, and 100 μM. Vehicle-treated cells served as the 0 μM control, and the final DMSO concentration was maintained at ≤0.1%. DLD-1, SW480, and fibroblasts were treated for 48 h, and viability was determined using the CCK-8 assay. Additional procedures are described in Supplementary Methods (Section S4.13).

### 4.14. Statistical Analysis

Quantitative data are presented as mean ± SEM from at least three independent biological experiments unless otherwise stated; animal experiments used six mice per group. Two-group comparisons used unpaired two-tailed Student’s t-tests. Comparisons involving three or more groups used one-way ANOVA followed by Dunnett’s test, except for Figure 4 and Figure 5, which used Tukey’s test. Longitudinal animal data were analyzed using two-way repeated-measures ANOVA with Greenhouse–Geisser correction. Tumor volume and relative tumor volume were additionally compared using separate one-way ANOVAs followed by Tukey’s test at each post-baseline time point, with adjustment within each time point but not across time points. RT-qPCR findings are presented descriptively without inferential statistical testing. Statistical significance was defined as *p* < 0.05. Full procedures are provided in Supplementary Methods (Section S4.14).

## 5. Conclusions

HST exerted apoptosis-associated antitumor effects in CRC cells and suppressed tumor growth in vivo. Co-treatment with HST at 0.3 mg/mL significantly reduced cell viability compared with 5-FU monotherapy in both DLD-1 and SW480 cells. These findings support further preclinical evaluation of HST as a potential complementary therapeutic strategy for CRC.

## Figures and Tables

**Table 1:** Serum biochemistry results for hepatic and renal functions.

Indicator (*n* = 6)	Control Group	HST 250 mg/kg/day	HST 1000 mg/kg/day
AST (IU/L)	78.0 ± 10.6	76.7 ± 7.1	87.5 ± 12.4
ALT (IU/L)	25.3 ± 2.1	24.8 ± 2.7	29.7 ± 4.4
BUN (mg/dL)	22.8 ± 2.6	24.2 ± 0.6	21.0 ± 0.7
CRE (mg/dL)	0.108 ± 0.010	0.105 ± 0.006	0.098 ± 0.007
UA (mg/dL)	3.75 ± 0.44	3.40 ± 0.54	2.07 ± 0.27 *

Note: Data are expressed as mean ± SEM (*n* = 6 mice per group). Statistical comparisons were performed using one-way ANOVA followed by Dunnett’s test. * *p* < 0.05 vs. control. AST, aspartate aminotransferase; ALT, alanine aminotransferase; BUN, blood urea nitrogen; CRE, creatinine; UA, uric acid; SEM, standard error of the mean; HST, Hangeshashinto.

**Table 2:** Composition of Hangeshashinto.

Crude Drugs	Scientific Names	Content (g, %)
Pinellia tuber	*Pinellia ternata* Breitenbach	5.0 g (25.6%)
Scutellaria root	*Scutellaria baicalensis* Georgi	2.5 g (12.8%)
Processed ginger	*Zingiber officinale* Roscoe	2.5 g (12.8%)
Glycyrrhiza	*Glycyrrhiza uralensis* Fischer	2.5 g (12.8%)
Jujube	*Ziziphus jujuba* Mill. var. *inermis* Rehder	2.5 g (12.8%)
Ginseng	*Panax ginseng* CA Meyer	2.5 g (12.8%)
Coptis rhizome	*Coptis japonica* Makino	1.0 g (5.1%)

Notes: Scientific names are based on *The Japanese Pharmacopoeia*, 18th Edition. Percentages indicate the proportion of each crude drug relative to the total formula weight.

**Table 3:** Primer sequences used for RT-qPCR analysis.

Gene	Forward Primer (5′→3′)	Reverse Primer (5′→3′)	Product Size (bp)	Function
*BCL2* (BCL-2)	CTTTGAGTTCGGTGGGGTCA	GGGCCGTACAGTTCCACAAA	134	Anti-apoptotic regulator
*BAX*	CCCGAGAGGTCTTTTTCCGAG	CCAGCCCATGATGGTTCTGAT	112	Proapoptotic effector (mitochondrial pathway)
*TNFRSF10A* (DR4)	TTGTCCTCACCCATGTCTCG	CAGGCTGTTGTTGAGGTTGC	138	Death receptor 4 (extrinsic apoptosis)
*TNFRSF10B* (DR5)	TTGAGCCACTGCTGTGTGAC	TCGGAGTTGCTGTTTCCACT	144	Death receptor 5 (extrinsic apoptosis)
*TP53* (p53)	GAGGTTGGCTCTGACTGTACC	GGTGTAGTGGATGGTGGTACAG	121	Tumor suppressor (transcriptional regulator)
*BBC3* (PUMA)	GACGACCTCAACGCACAGTA	AGGAGTCCCATGATGAGATTGT	125	p53-inducible pro-apoptotic gene
*GAPDH*	AAGGTGAAGGTCGGAGTCAA	AATGAAGGGGTCATTGATGG	130	Internal control (housekeeping gene)

Bp, base pairs. Note: All primers were produced by Thermo Fisher Scientific (Waltham, MA, USA). PCR products were confirmed by melting-curve analysis and agarose gel electrophoresis.

## Data Availability

The data supporting the findings of this study are available within the article and its Supplementary Materials.

## Supplementary Materials

The following supporting information can be downloaded at: https://www.mdpi.com/article/10.3390/ijms27209310/s1.

## Abbreviations

The following abbreviations are used in this manuscript:

2^−ΔΔCt	Relative quantification method
5-FU	5-fluorouracil
AIF	Apoptosis-inducing factor
Annexin V–FITC	Fluorescein isothiocyanate-conjugated Annexin V
*BAX*	BCL2-associated X protein
BCL-2	B-cell lymphoma 2
CCK-8	Cell Counting Kit-8
CRC	Colorectal cancer
DAPI	4′,6-Diamidino-2-phenylindole
DMSO	Dimethyl sulfoxide
DR4	Death receptor 4
DR5	Death receptor 5
EndoG	Endonuclease G
FBS	Fetal bovine serum
*GAPDH*	Glyceraldehyde-3-phosphate dehydrogenase
H&E	Hematoxylin and eosin
HPLC	High-performance liquid chromatography
HST	Hangeshashinto
IC_50_	Half-maximal inhibitory concentration
LC/MS	Liquid chromatography–mass spectrometry
PI	Propidium iodide
PK/PD	Pharmacokinetics/pharmacodynamics
PUMA	P53 upregulated modulator of apoptosis
RT-qPCR	Reverse transcription–quantitative polymerase chain reaction
SEM	Standard error of the mean
UA	Uric acid
z-VAD-FMK	Pan-caspase inhibitor
