# Supplementary material for: Hangeshashinto Induces Apoptosis and Suppresses Colorectal Cancer Growth In Vitro and In Vivo
Source: Int J Mol Sci. 2026 Oct 20;27(20):9310. doi: 10.3390/ijms27209310 (PMC13705497; doi:10.3390/ijms27209310)
Supplement: Supplementary file 1 [file ijms-27-09310-s001.zip › ijms-4603952-supplementary.pdf]

Supplementary Materials

Contents (Index)

Supplementary Table S1. Quantitative component profile of HST and estimated constituent concentrations at 1 mg/mL HST.

Supplementary Table S2. Growth inhibition profiles and estimated IC_{50} values of ten representative constituents of Hangeshashinto across colorectal cancer cell lines and normal fibroblasts.

Supplementary Table S3. Sources of cells, reagents, media, antibodies, and chemicals used in this study.

Supplementary Figure S1. Three-dimensional HPLC (UV) chromatogram of the HST extract.

Supplementary Figure S2. RT-qPCR analysis of apoptosis-related genes in DLD-1 and SW480 cells treated with HST.

Supplementary Figure S3. Screening of ten representative constituents of HST in DLD-1, SW480, and Fibro cells.

Supplementary Methods: Sections S4.6–S4.14

Supplementary Table S1. Quantitative component profile of HST and estimated constituent concentrations at 1 mg/mL HST.

No.	Compound	Herbal origin	Retention time (min)	Content (mg/g extract)	Estimated concentration at 1 mg/mL HST (μM)	Isolated-compound test range (μM)	Reference standard/Source
1	Baicalin	Scutellariae Radix	15.8	16.8	37.64	12.5–100	JP18/Tsumura
2	Wogonin	Scutellariae Radix	20.6	1.9	6.68	12.5–100	JP18/Tsumura
3	Glycyrrhizin	Glycyrrhizae Radix	24.7	5.6	6.80	12.5–100	JP18/Tsumura
4	Berberine	Coptidis Rhizoma	28.2	0.9	2.68	12.5–100	JP18/Tsumura
5	6-Gingerol	Zingiberis Rhizoma Processum	33.5	0.5	1.70	12.5–100	JP18/Tsumura
6	Baicalein	Scutellariae Radix	18.1	Trace	<1.11	12.5–100	Confirmed by LC/MS
7	Wogonoside	Scutellariae Radix	19.8	Trace	<0.65	Not tested	Confirmed by LC/MS
8	Liquiritin	Glycyrrhizae Radix	13.5	Trace	<0.72	12.5–100	Confirmed by LC/MS
9	Palmatine	Coptidis Rhizoma	29.1	Trace	<0.85	Not tested	Confirmed by LC/MS
10	Magnoflorine	Pinelliae Tuber	12.3	Trace	<0.88	Not tested	Confirmed by LC/MS

Notes: Quantification was performed by 3D-HPLC (Tsumura analytical method) using UV detection (254 nm or compound-specific wavelengths) and calibration with JP18 reference standards. Trace indicates a content of <0.3 mg/g extract.

Estimated concentrations were calculated from the reported constituent contents at an HST extract concentration of 1 mg/mL using: concentration (μM) = content (mg/g extract) × 1000 / molecular weight (g/mol). Molecular weights used were 446.36 (baicalin), 284.26 (wogonin), 822.94 (glycyrrhizin,

expressed as glycyrrhizic acid), 336.36 (berberine), 294.39 (6-gingerol), 270.24 (baicalein), 460.39 (wogonoside), 418.40 (liquiritin), 352.41 (palmatine), and 342.41 (magnoflorine). Berberine, palmatine, and magnoflorine were calculated on a cation basis, excluding counterions. Values are approximate; values for trace constituents represent estimated upper limits.

These estimates are nominal extract-equivalent concentrations, not measured concentrations in the culture medium or cells. Isolated compounds were tested at 12.5, 25, 50, and 100 μM. "Not tested" indicates that the constituent was not included in the isolated-compound screening. Ginsenosides Rg1 and Rb1 and isoliquiritigenin were included in the screening, but their extract-equivalent concentrations could not be estimated because their contents were not reported in this table. Activity at concentrations exceeding the estimated extract-equivalent concentrations does not by itself establish a substantial contribution to the effects of the whole HST extract.

Abbreviations: HST, Hangeshashinto; 3D-HPLC, three-dimensional high-performance liquid chromatography; UV, ultraviolet; JP18, *Japanese Pharmacopoeia*, 18th edition; LC/MS, liquid chromatography–mass spectrometry.

Supplementary Table S2. Growth inhibition profiles and estimated IC_{50} values of ten representative constituents of Hangeshashinto across colorectal cancer cell lines and normal fibroblasts.

Compound	DLD-1 IC_{50} (µM)	SW480 IC_{50} (µM)	Fibro IC_{50} (µM)	Inhibition at 100 µM (%) DLD-1 / SW480 / Fibro	Major observation
Baicalein	33.1	<12.5	>100 (ND)	67.3 / 90.7 / −10.0	Strong inhibition in both CRC lines; SW480 was most sensitive; no inhibition in Fibro.
Wogonin	>100 (ND)	<12.5	>100 (ND)	44.7 / 75.7 / 39.8	Marked inhibition in SW480; DLD-1 and Fibro did not reach 50% inhibition.
Baicalin	86.0	90.6	>100 (ND)	52.0 / 51.9 / 0.3	Gradual inhibition in both CRC lines; Fibro remained unaffected.
Berberine	>100 (ND)	26.2	>100 (ND)	−1.8 / 69.4 / 0.7	SW480-selective inhibition; no inhibitory response in DLD-1 or Fibro.
6-Gingerol	>100 (ND)	>100 (ND)	>100 (ND)	3.6 / 14.3 / −10.0	No 50% inhibition in any cell type within the tested range.
Ginsenoside Rg1	>100 (ND)	>100 (ND)	>100 (ND)	7.5 / 0.3 / −12.1	No 50% inhibition; viability remained near or above control.
Ginsenoside Rb1	>100 (ND)	>100 (ND)	>100 (ND)	12.9 / 2.0 / −14.8	No 50% inhibition; only mild DLD-1 reduction at 100 µM.
Isoliquiritigenin	96.4	>100 (ND)	>100 (ND)	53.4 / −21.3 / 44.4	DLD-1 reached ~50% viability near 100 µM; Fibro decreased but did not reach IC50.

Compound	DLD-1 IC_{50} (μM)	SW480 IC_{50} (μM)	Fibro IC_{50} (μM)	Inhibition at 100 μM (%) DLD-1 / SW480 / Fibro	Major observation
Liquiritin	>100 (ND)	>100 (ND)	>100 (ND)	−1.1 / −3.8 / −25.0	No suppression observed; SW480 and Fibro responses were at or above control.
Glycyrrhizic acid	>100 (ND)	>100 (ND)	>100 (ND)	−19.2 / −24.5 / −10.1	No suppression observed; viability remained at or above control.

Notes:

• Data are based on three independent biological experiments (n = 3), with cell viability normalized to the overall vehicle-control mean for each cell model and compound (= 100%).

• Numeric IC_{50} values are reported only when the confirmed mean dose–response crossed 50% viability within the tested concentration range (12.5–100 μM). If viability was already <50% at 12.5 μM, IC_{50} is reported as <12.5 μM; if viability remained >50% at 100 μM, IC_{50} is reported as >100 μM (ND).

• For curves crossing 50% viability within the tested range, IC_{50} values were estimated by nonlinear sigmoidal inhibitory-response fitting with the untreated control normalized to 100%.

• The inhibition column reports 100 − mean viability (%) at 100 μM in the order DLD-1 / SW480 / Fibro; negative values indicate viability above the vehicle-control mean.

• ND indicates that 50% inhibition was not achieved within the tested concentration range.

Supplementary Table S3. Sources of cells, reagents, media, antibodies, and chemicals used in this study.

Category	Reagent/Material	Supplier (City, Country)	Catalog No./Note
Cell lines	DLD-1 (human colorectal cancer cell line)	JCRB Cell Bank, Osaka, Japan	JCRB9094; Cellosaurus: CVCL_0248; RRID:CVCL_0248
	SW-480/CMV-Luc (SW480-derived luciferase-expressing colorectal cancer cell line; referred to as "SW480" in this study)	JCRB Cell Bank, Osaka, Japan	NIHS0738; Cellosaurus: CVCL_4Y13; RRID:CVCL_4Y13
	Human rectal fibroblasts (HRecF)	ScienCell Research Laboratories, Carlsbad, CA, USA	Cat. No. 2960
	Adult human dermal fibroblasts (Fibro; primary cells)	Cell Applications, Inc., San Diego, CA, USA; supplied	Cat. No. CA106K05a

		through Toyobo Co., Ltd., Osaka, Japan	
	CCD 841 CoN (non-malignant human colonic cell line; referred to as CCD841CoN in this study)	American Type Culture Collection (ATCC), Manassas, VA, USA	Cat. No. CRL-1790; Cellosaurus: CVCL_2871; RRID:CVCL_2871
Culture media & reagents	RPMI-1640 medium	Gibco, Grand Island, NY, USA	11875-093
	F-Medium (for dermal fibroblasts)	Kurabo Industries Ltd., Osaka, Japan	F2100
	Fetal bovine serum (FBS)	Gibco	16000-044
	Penicillin–Streptomycin (P/S)	Gibco	15140-122
	Trypsin-EDTA (0.25%)	Gibco	25200-056
Cell viability & apoptosis assays	Cell Counting Kit-8 (CCK-8)	Dojindo Laboratories, Kumamoto, Japan	CK04-11

	Hoechst 33258 staining solution	Dojindo Laboratories, Kumamoto, Japan	H341
	Dead Cell Apoptosis Kit with Annexin V FITC & Propidium Iodide	Thermo Fisher Scientific, Waltham, MA, USA	V13242
	Caspase-3 Activity Assay Kit	Cell Signaling Technology, Danvers, MA, USA	#5723
	Cleaved Caspase-3 (Asp175) (5A1E) Rabbit Monoclonal Antibody	Cell Signaling Technology	#9664S
	Anti-rabbit IgG (H+L), F(ab')2 Fragment (Alexa Fluor® 594 Conjugate)	Cell Signaling Technology	#8889
	Z-VAD-FMK (caspase inhibitor)	Promega, Madison, WI, USA	G7231
Other reagents	Propidium iodide (PI)	FUJIFILM Wako Pure Chemical Industries, Osaka, Japan	163-26261
	DAPI/VECTASHIELD Mounting Medium	Vector Laboratories, Newark, CA, USA	H-1200

	Paraformaldehyde (3.5%)	Muto Chemicals Co., Tokyo, Japan	N/A
	Glass-bottom dish (35 mm)	Matsunami Glass Ind., Ltd., Osaka, Japan	D11130H
Molecular assays	RNeasy Plus Mini Kit	QIAGEN, Hilden, Germany	74134
	ReverTra Ace qPCR RT Master Mix with gDNA Remover	Toyobo Co., Ltd., Osaka, Japan	FSQ-301
	KOD SYBR qPCR Mix	Toyobo Co., Ltd., Osaka, Japan	QKD-201
General chemicals	Dimethyl sulfoxide (DMSO)	Sigma-Aldrich	D2650
	Giemsa stain solution	FUJIFILM Wako	054-00522
	HST extract (spray-dried extract)	Tsumura & Co., Tokyo, Japan	Lot 2210014010

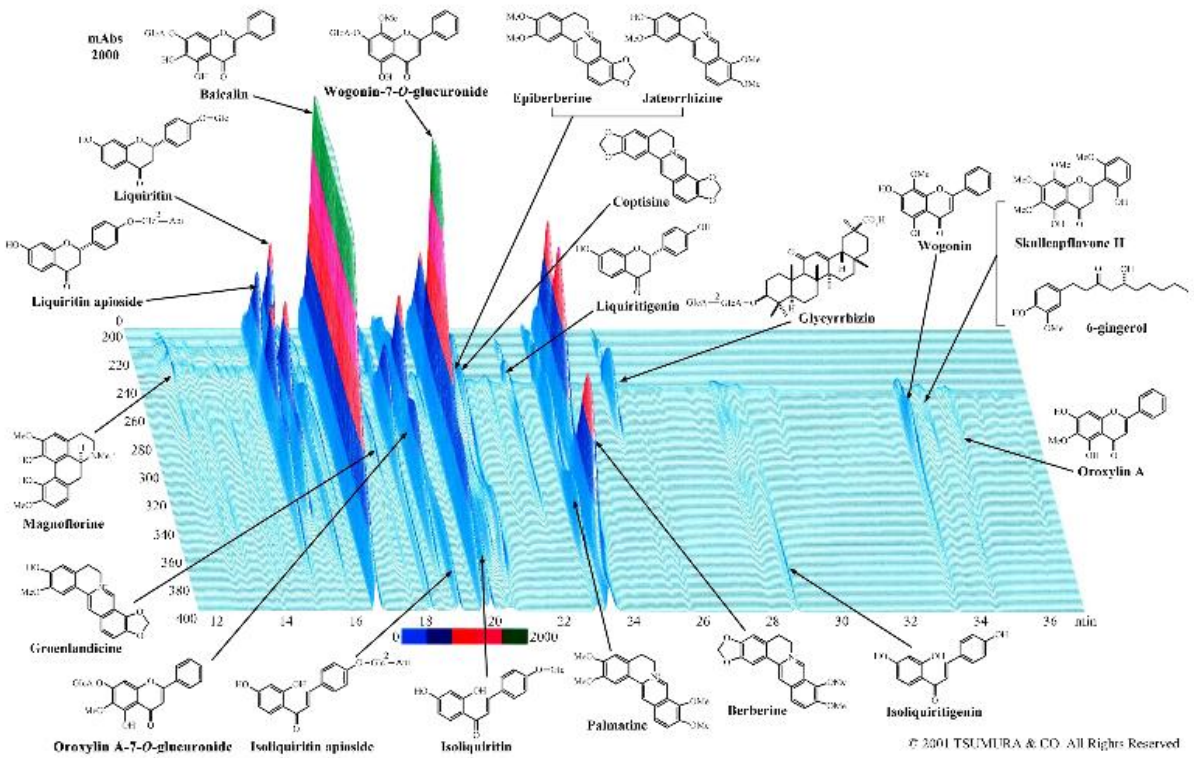

Supplementary Figure S1.

Three-dimensional HPLC (UV) chromatogram of the HST extract. Representative peaks corresponding to major constituents of HST are shown. Quantitative marker contents (mg/g, w/w) are summarized in Supplementary Table S1. Peak assignments were performed by

Tsumura & Co. based on reference standards and previous reports. This image is reproduced with permission from Tsumura & Co. ©

Tsumura & Co.

Abbreviations: HST, Hangeshashinto; HPLC, high-performance liquid chromatography; UV, ultraviolet.

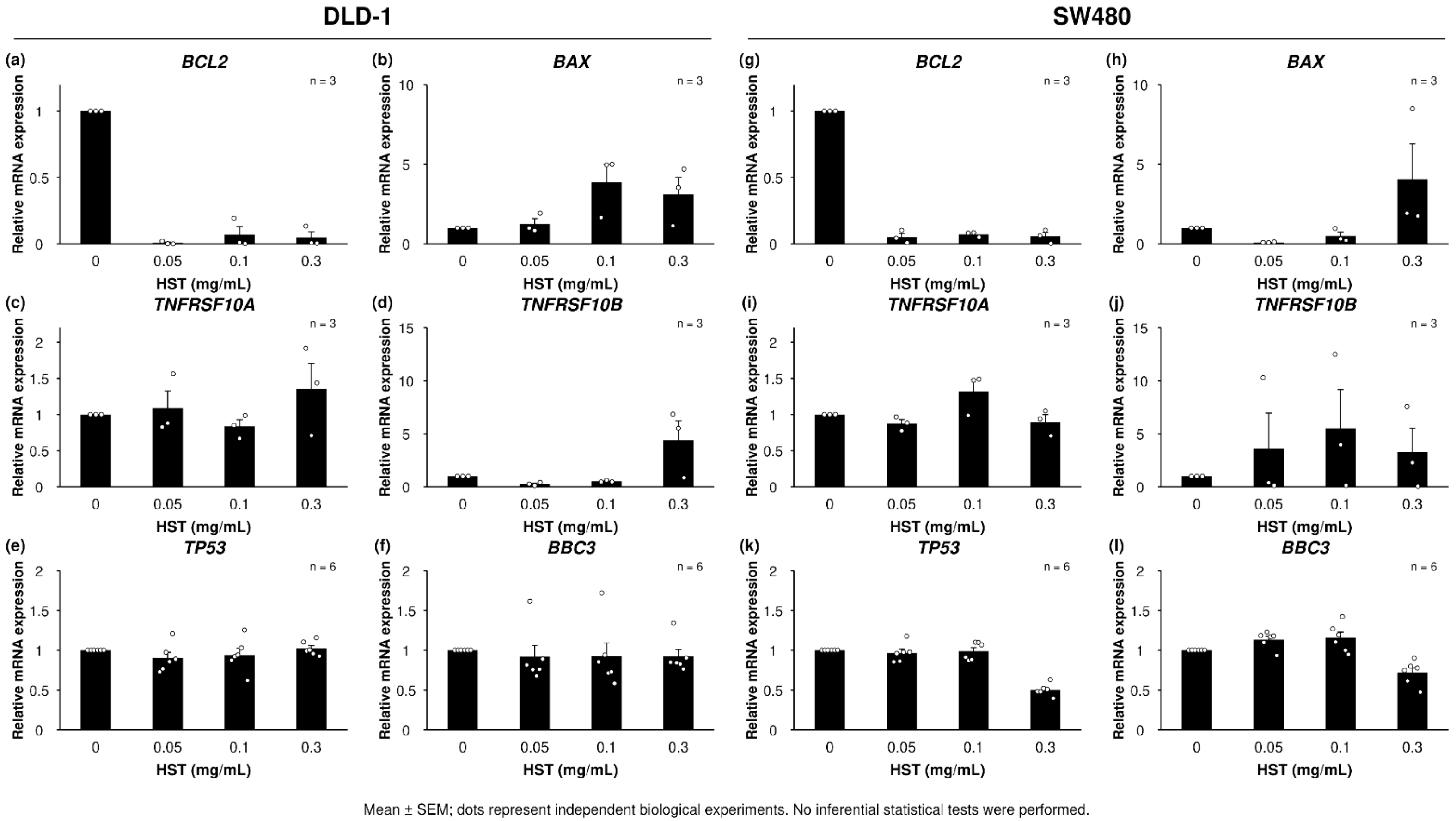

Supplementary Figure S2.

RT-qPCR analysis of apoptosis-related genes in DLD-1 and SW480 cells treated with HST. (a–f) DLD-1 cells; (g–l) SW480 cells. Relative

mRNA expression of *BCL2*, *BAX*, *TNFRSF10A*, *TNFRSF10B*, *TP53*, and *BBC3* after 48 h of treatment with HST (0, 0.05, 0.1, and 0.3 mg/mL). Expression levels were normalized to *GAPDH* and calculated using the $2^{-\Delta\Delta Ct}$ method. Data are presented as mean ± SEM from three independent experiments (n = 3) for *BCL2*, *BAX*, *TNFRSF10A*, and *TNFRSF10B*, and six independent experiments (n = 6) for *TP53* and *BBC3*. Dots represent independent biological experiments. These exploratory data are presented descriptively without inferential statistical testing.

Abbreviations: HST, Hangeshashinto; RT-qPCR, reverse transcription–quantitative polymerase chain reaction; mRNA, messenger RNA; SEM, standard error of the mean; Ct, cycle threshold; *BCL2*, B-cell lymphoma 2; *BAX*, BCL2-associated X protein; *TNFRSF10A*, tumor necrosis factor receptor superfamily member 10A (DR4, death receptor 4); *TNFRSF10B*, tumor necrosis factor receptor superfamily member 10B (DR5, death receptor 5); *TP53*, tumor protein p53; *BBC3*, BCL2-binding component 3 (PUMA, p53 upregulated modulator of apoptosis); *GAPDH*, glyceraldehyde-3-phosphate dehydrogenase.

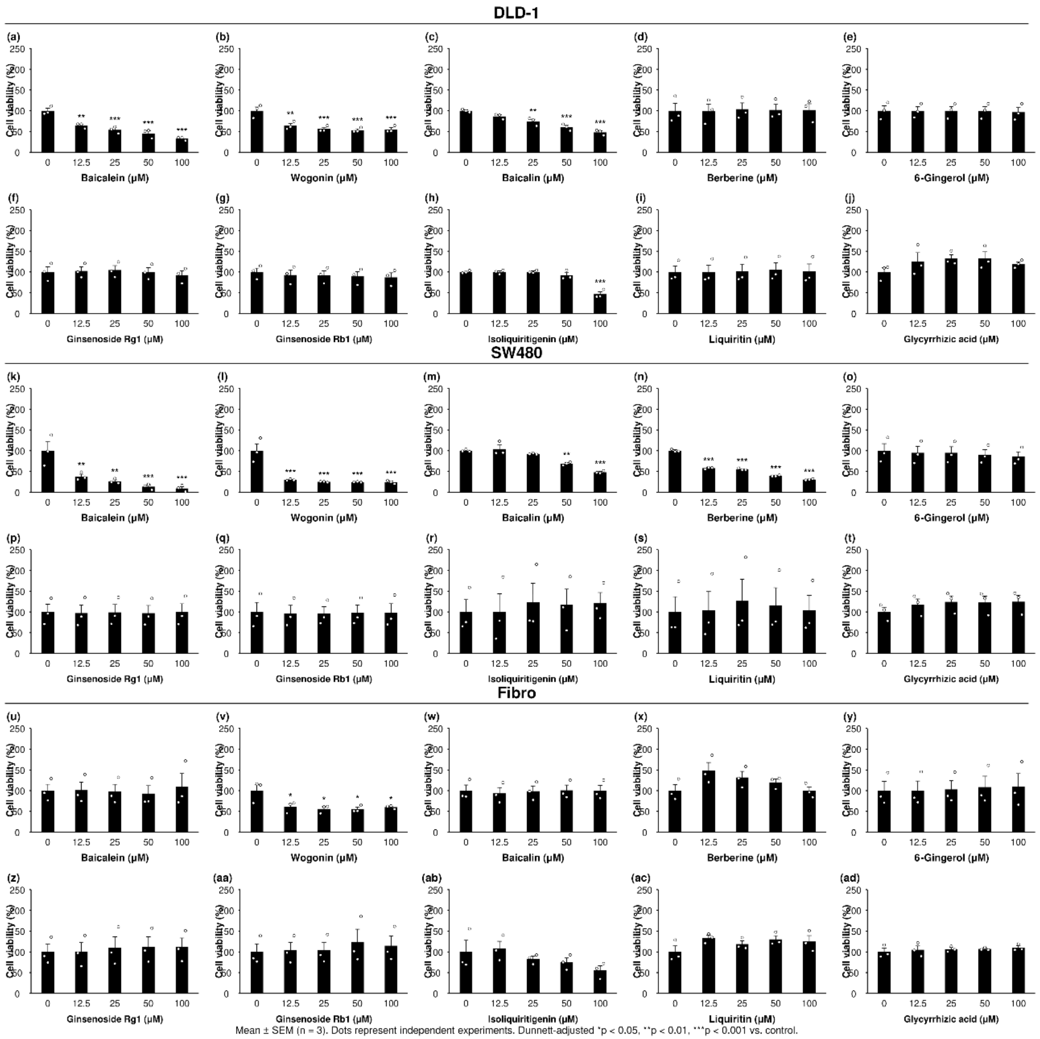

Mean ± SEM (n = 3). Dots represent independent experiments. Dunnett-adjusted *p < 0.05, **p < 0.01, ***p < 0.001 vs. control.

Supplementary Figure S3.

Screening of ten representative constituents of HST in DLD-1, SW480, and Fibro cells. (a–j) DLD-1; (k–t) SW480; (u–ad) Fibro. Cells were treated with each compound (0–100 μM) for 48 h, and cell viability was assessed using the CCK-8 assay (absorbance at 450 nm). Data are presented as mean ± SEM from three independent biological experiments (n = 3). Dots represent independent biological experiments. Statistical significance was evaluated using one-way ANOVA followed by Dunnett's test.

$*p < 0.05$, $**p < 0.01$, $***p < 0.001$ vs. vehicle control.

Abbreviations: HST, Hangeshashinto; CCK-8, Cell Counting Kit-8; SEM, standard error of the mean; ANOVA, analysis of variance; Fibro, adult human dermal fibroblasts.

Supplementary Methods

These Supplementary Methods provide detailed procedural descriptions corresponding to Sections 4.6–4.14 of the main text. All experiments were conducted under the conditions described in Section 4.1. Unless otherwise stated, data were derived from at least three independent biological replicates.

S4.6 Cell viability assay (CCK-8)

For the CCK-8 assay, DLD-1, SW480, and CCD841CoN cells were seeded into 96-well plates at 5×10^3 cells per well, and HRecF and dermal fibroblasts (Fibro) were plated at 3×10^3 cells per well. After preincubation for 24 h under standard conditions (37°C, 5% CO_2), the culture medium was replaced with fresh medium containing HST at 0, 0.1, 0.3, 0.5, or 1.0 mg/mL. For HST treatments, untreated control wells containing complete medium without DMSO were included on every plate. Cells were exposed for 48 h, after which 10 µL of CCK-8 reagent was added to each well, and the plates were incubated for an additional 2 h at 37°C. Absorbance at 450 nm was then recorded. A cell-free blank containing medium plus CCK-8 was used to correct for background. For Figure 2a–e, cell viability was expressed as a percentage of the mean untreated-control value within each independent experiment (= 100%) after blank subtraction.

S4.7 Colony formation assay

Clonogenic survival was evaluated in DLD-1 and SW480 cells. Briefly, cells were seeded into six-well plates at 600 cells per well and allowed to adhere for 24 h. They were then cultured in medium containing HST at 0, 0.1, 0.3, or 1.0 mg/mL for 14 days, with the medium being renewed every 2–3 days. Following incubation, colonies were fixed with 3.5% paraformaldehyde at room temperature for 30 min, stained with Giemsa solution, washed with water, and air-dried. Plates were imaged under uniform lighting, and colonies were counted using ImageJ software (version 1.53) with a fixed threshold and a minimum area filter to exclude background staining and small artifacts.

Total number of colonies for each well was recorded, and the surviving fraction was calculated by dividing the colony count in the HST-treated well by the mean colony count of the untreated control wells and multiplying the quotient by 100.

S4.8 Apoptosis assays

Apoptotic responses to HST were assessed using complementary approaches: annexin V/propidium iodide (PI) flow cytometry, caspase-3 activity measurement, immunofluorescent detection of cleaved caspase-3, and nuclear morphology analysis. All assays were conducted using DLD-1 and SW480 cells under the same culture conditions described earlier.

S4.8.1 Annexin V/PI flow cytometry

For annexin V/PI staining, DLD-1 and SW480 cells were treated with HST at 0, 0.1, 0.3, or 1.0 mg/mL for 48 h, with medium alone serving as the untreated control. After treatment, floating and adherent cells were collected, washed with ice-cold phosphate-buffered saline (PBS), and resuspended in 1× annexin binding buffer at approximately 1×10^6 cells/mL. Annexin V–FITC and propidium iodide (PI, 5 µg/mL) were added according to the Annexin V–FITC/Dead Cell Apoptosis Kit protocol, and the samples were incubated for 15 min at room temperature in the dark before adding additional binding buffer. Acquisition of flow cytometry data was performed on a BD FACSCelesta™ instrument at 488 nm excitation, with at least 1×10^4 events collected for each sample. Nonstained and single-stained controls were used for compensation. Debris and doublets were excluded on forward and side scatter plots, and cells were classified into

viable, early apoptotic, late apoptotic, and necrotic fractions based on annexin V and PI signals. Data were analyzed using FlowJo software, version 10.8.1 (BD Biosciences), with identical gating applied to all groups within an experiment.

S4.8.2 Caspase-3 activity assay

Caspase-3 activation was quantified using a commercial Caspase-3 Activity Assay Kit. Briefly, DLD-1 and SW480 cells were treated with HST at 0, 0.1, 0.3, 0.5, or 1.0 mg/mL for 24 h. After treatment, floating and adherent cells were harvested, washed in cold PBS, and lysed on ice in the kit lysis buffer for 10 min. Lysates were then clarified via centrifugation at 12,000 × *g* for 10 min at 4°C, and the supernatants were transferred to new tubes. Reaction mixtures were prepared by combining DTT-containing assay buffer with the fluorogenic substrate Ac-DEVD-AMC to a final volume of 100 µL per well and then adding equal volumes of supernatant. After incubation at 37°C in the dark for 1 h, fluorescence was measured at excitation and emission wavelengths of 380 and 450 nm, respectively, using a multimode microplate reader. A preliminary time-course experiment (12–48 h) showed that most conditions yielded maximal caspase-3 activity at 24 h. Therefore, this time point was used for the main analysis. Caspase-3 activity was expressed as the ratio of fluorescence signals measured in relative fluorescence units (RFU) to the untreated control (set as 1.0).

S4.8.3 Immunofluorescence for cleaved caspase-3

For immunofluorescence, DLD-1 and SW480 cells were plated on 35 mm glass-bottom dishes at 1×10^4 cells in 200 µL of medium and incubated for 24 h. The cells were then treated with HST at 0, 0.1, 0.3, or 1.0 mg/mL for 48 h (medium only as the untreated control).

After treatment, the cells were washed with PBS, fixed in 3.5% paraformaldehyde at room temperature for 15 min, rinsed, and permeabilized with 0.1% Triton X-100 in PBS for 10 min. Non-specific binding was blocked with PBS containing bovine serum albumin and normal goat serum. Samples were incubated overnight at 4°C with a primary antibody against cleaved caspase-3 (Asp175), washed, and then incubated at room temperature with an Alexa Fluor-conjugated secondary antibody in the dark. Nuclei were counterstained and mounted with DAPI-containing medium. Images were captured using a BZ-700X fluorescence microscope using identical exposure and gain settings for all conditions within each experiment.

S4.8.4 Nuclear morphology staining

Nuclear morphology was examined via Hoechst 33258 staining. Briefly, DLD-1 and SW480 cells were seeded onto 35 mm glass-bottom dishes, treated with HST at 0, 0.1, 0.3, or 1.0 mg/mL for 48 h, washed with PBS, and fixed in 3.5% paraformaldehyde at 4°C for 15 min. After washing, the cells were incubated with Hoechst 33258 (1 µg/mL) solution for 15 min at room temperature and imaged using a BZ-700X microscope. Chromatin condensation and nuclear fragmentation were used as morphological indicators of apoptosis, and exposure settings were kept constant across groups.

S4.9 Pharmacological test for caspase dependence

S4.9.1 z-VAD-FMK concentrations and preincubation

To determine the nontoxic concentration of z-VAD-FMK, DLD-1 and SW480 cells were seeded into 96-well plates at 5 × 10^3 cells per well and preincubated for 24 h. Cells were then treated with z-VAD-FMK at 0, 10, 30, 50, or 100 μM for 48 h. The vehicle consisted of culture medium containing 0.1% DMSO. Cell viability was assessed via the CCK-8 assay. Given that z-VAD-FMK alone did not reduce viability across this range, 10 μM was selected as the working concentration for subsequent rescue experiments. For all groups, the final DMSO concentration was kept at or below 0.1%.

S4.9.2 Rescue of HST-induced growth inhibition

For rescue experiments, cells were preincubated with 10 μM z-VAD-FMK for 30 min and subsequently treated with HST at 0.1 or 0.3 mg/mL for 48 h under the same conditions as the viability assays. Cell viability was then evaluated using the CCK-8 assay to assess whether z-VAD-FMK attenuated HST-induced growth inhibition.

S4.10 Reverse transcription–quantitative PCR (RT-qPCR) analysis

RT-qPCR was performed to assess transcriptional changes in apoptosis-related genes in DLD-1 and SW480 cells following HST treatment. Briefly, cells were exposed to 0, 0.05, 0.1, or 0.3 mg/mL HST for 48 h in RPMI-1640 medium supplemented with 10% FBS and 1% penicillin–streptomycin at 37°C in a humidified 5% CO_2 atmosphere. When cultures reached approximately 80%–90% confluence, total RNA was extracted using the RNeasy Plus Mini Kit (Cat. No. 74134; QIAGEN, Hilden, Germany) according to the manufacturer's

protocol. Thereafter, 1 µg of total RNA was reverse-transcribed into cDNA using the ReverTra Ace qPCR RT Master Mix with gDNA Remover (Toyobo Co., Ltd., Osaka, Japan; FSQ-301).

qPCR was performed using KOD SYBR qPCR Mix (Toyobo Co., Ltd., Osaka, Japan) on an Applied Biosystems 7500 Fast Real-Time PCR System. Each 20 µL reaction contained 10 µL Master Mix, 2 µL cDNA, and 0.4 µM primers. Thermal cycling was conducted as follows: 95°C for 10 min, followed by 40 cycles of 95°C for 15 s and 60°C for 1 min. Target genes included *BCL2* (BCL-2), *BAX*, *TNFRSF10A* (DR4), *TNFRSF10B* (DR5), *TP53* (p53), and *BBC3* (PUMA) (Table 3). *GAPDH* served as the internal control. Gene expression was calculated using the ΔΔCt method: ΔCt = Ct_target − Ct_GAPDH, and ΔΔCt = ΔCt_sample − ΔCt_control. Fold-change values were expressed as $2^{-\Delta\Delta Ct}$, with the untreated control set to 1.0.

S4.11 Combination treatment with 5-fluorouracil (5-FU)

For the monotherapy experiments shown in Figure 5a, DLD-1, SW480, HRecF, Fibro, and CCD841CoN cells were treated with 5-FU at 0, 10, 30, 50, or 100 µM for 48 h. Cell viability was assessed using the CCK-8 assay; only the untreated control and 30 µM results are displayed in Figure 5a. Combination assays were performed to evaluate whether HST altered cellular responses to 5-FU. Briefly, DLD-1 and SW480 cells were seeded at 5×10^3 cells per well, allowed to attach for 24 h, and then treated for 48 h with 5-FU (30 µM), HST (0.1 or 0.3 mg/mL), or both. Viability was then assessed using the CCK-8 assay as described in Section S4.6. For Figure 5b, c, viability was

normalized to the overall untreated-control mean within each cell line (= 100%; n = 3 for DLD-1 and n = 4 for SW480). Comparisons between 5-FU alone and HST co-treatment groups were performed as described in Section S4.14; formal synergy analysis was not performed.

S4.12 *In vivo* subcutaneous xenograft tumor model

S4.12.1 Animals, grouping, and oral administration

A total of 34 female BALB/c-nu nude mice were obtained from The Jackson Laboratory Japan, Inc. (Yokohama, Japan) and housed under controlled conditions (12 h light/dark cycle, approximately 23°C, and ad libitum access to food and water). After at least one week of acclimatization, 5×10^6 DLD-1 cells suspended in 0.2 mL of a 1:2 Matrigel/HBSS mixture were injected subcutaneously into the dorsal flank. All procedures were approved by the Institutional Animal Care and Use Committee of Toyo University (Approval No. 2024-14; approved on 27 March 2024). Humane endpoints included intolerable distress or ≥20% body-weight loss.

On day 14 after tumor inoculation, 16 mice with tumor volumes below 10 mm^3 were excluded before treatment. The remaining 18 mice were allocated to three groups (n = 6 mice per group): vehicle control, HST 250 mg/kg/day, and HST 1000 mg/kg/day. Animals were ranked according to baseline body weight and tumor volume and arranged into six blocks of three mice with similar measurements. Within each block, random numbers generated using the RAND function in Microsoft Excel were used to assign one mouse to each group. Mean

body weight and tumor volume were then checked across groups. The entire random allocation procedure was repeated several times, and a grouping judged to provide satisfactory balance in these baseline variables was selected. After treatment initiation, there were no deaths, exclusions, or missing observations, and no animals or data points were excluded from the analyses. The group size was determined with reference to a previous DLD-1 xenograft study that used six mice per group [28]; no a priori statistical sample size calculation was performed. HST or vehicle (distilled water) was administered orally twice daily for 14 days, with the total daily HST dose divided into two administrations. The HST doses were selected with reference to a previous animal study using oral HST at 250–1000 mg/kg [29]. The 14-day treatment period was informed by a previous study using approximately two weeks of oral hochuekkito administration in tumor-bearing mice [30]. Twice-daily administration was chosen with reference to divided dosing in clinical Kampo use. Following the 14-day treatment period, mice underwent a 7-day washout period before euthanasia and necropsy. This washout period was included to monitor tumor growth during the short interval following treatment cessation.

S4.12.2 Tumor volume and body weight measurements

Tumor dimensions were measured every 3–4 days using a digital caliper, with body weights recorded concurrently. Tumor volume (V) was calculated as $V = (\text{length} \times \text{width}^2)/2$, where length and width represent the longest and shortest diameters, respectively. Tumor dimensions were measured by an investigator blinded to treatment allocation, with animals identified by individual identification numbers. Treatment administration and tumor measurements were performed by different investigators. Data are presented as mean ± standard error

of the mean (SEM).

S4.12.3 Blood collection, euthanasia, serum biochemistry, and tumor histology

Following the 7-day washout period, mice were anesthetized with 2% inhaled isoflurane. The absence of a response to a pinch stimulus was confirmed before laparotomy. Isoflurane anesthesia was maintained during terminal blood collection from the caudal vena cava, with approximately 1 mL collected per mouse depending on the individual animal. Exsanguination was followed by cervical dislocation without recovery from anesthesia. Death was confirmed by the absence of respiration, heartbeat, and response to a pinch stimulus. Tumors were subsequently excised.

Serum biochemical parameters, including alanine aminotransferase, aspartate aminotransferase, blood urea nitrogen, creatinine, and uric acid, were quantified using standard clinical chemistry assays. These parameters were evaluated based on intergroup comparisons rather than predefined reference ranges.

Tumor tissues were fixed in 10% neutral-buffered formalin, embedded in paraffin, and sectioned at 4 μm. Sections were stained with hematoxylin and eosin (H&E) for histopathological evaluation. Cleaved caspase-3 staining in tumor tissues was assessed by immunohistochemistry using an anti-cleaved caspase-3 antibody (Cell Signaling Technology, Danvers, MA, USA; #9664S; 1:200). Histopathological evaluation of H&E-stained tumor sections and quantification of cleaved caspase-3 staining were performed with knowledge of the treatment assignments and were not blinded.

S4.13 Screening of isolated constituents

To investigate the contribution of major phytochemicals to the cytotoxic activity of HST, a 48 h viability screening was performed using DLD-1, SW480, and Fibro cells. Accordingly, 10 representative constituents, namely baicalein, wogonin, baicalin, berberine, 6-gingerol, ginsenoside Rg1, ginsenoside Rb1, isoliquiritigenin, liquiritin, and glycyrrhizic acid, were prepared as DMSO stocks and diluted to 0, 12.5, 25, 50, and 100 μM. Final DMSO concentrations were ≤0.1% for all groups. Cells were seeded into 96-well plates (5×10^3 cells/well for DLD-1/SW480; 3×10^3 cells/well for Fibro), preincubated for 24 h, and then treated for 48 h. Cell viability was assessed using the CCK-8 assay and expressed as a percentage of the overall mean vehicle-control value across the three independent experiments (= 100%).

S4.14 Statistical analysis

Data are presented as mean ± SEM from at least three independent biological experiments unless otherwise stated; animal experiments used six mice per group. For Figure 5, technical replicates were averaged within each experiment (n = 3 for panels a, b; n = 4 for panel c). Analyses used GraphPad Prism 10.1.1 (GraphPad Software, San Diego, CA, USA); reanalyses of Figures 2a–e, h, 3c–f, 4a–d, 5b, c, Supplementary Figure S3, and animal data used Python with NumPy and SciPy 1.17.0.

Two-group comparisons used unpaired two-tailed Student's t-tests. Multiple-group comparisons used one-way ANOVA with Dunnett's

test, except for Figures 4 and 5, which used Tukey's test. Dunnett adjustment was applied separately within each cell model and assay, or each cell model and compound for Supplementary Figure S3, without adjustment across panels. Tukey's test covered all pairwise comparisons among five groups in Figure 4a, b and six groups in Figures 4c, d and 5b, c. Figure 5a included the control and all four 5-FU concentrations; only control-versus-30 μM comparisons are displayed. RT-qPCR data were descriptive, without inferential testing. Longitudinal animal data were analyzed using two-way repeated-measures ANOVA, with group as the between-subject factor and time as the within-subject factor; Greenhouse–Geisser correction was applied to time and group-by-time effects. Relative tumor volume was calculated by dividing each mouse's tumor volume by its baseline value, whereas body weight changes were calculated by subtracting its baseline weight; baseline values were excluded from these analyses. Exploratory comparisons of tumor volume and relative tumor volume used separate one-way ANOVAs followed by Tukey's test at each post-baseline time point, assuming normally distributed errors and equal variances. Adjustment covered all three pairwise comparisons within each time point, without adjustment across time points. Comparisons of body weight and body weight changes with controls at each post-baseline time point used two-sided Welch's t-tests with Holm correction across 12 comparisons per outcome (two HST groups × six time points). Serum biochemistry, tumor area ratios, and cleaved caspase-3-positive areas used one-way ANOVA with two-sided Dunnett's tests versus controls. Statistical significance was defined as $p < 0.05$ (*$p < 0.05$, **$p < 0.01$, ***$p < 0.001$).
